# Supplementary material for: The Mla system of diderm Firmicute Veillonella parvula reveals an ancestral transenvelope bridge for phospholipid trafficking
Source: Nat Commun. 2023 Nov 23;14:7642. doi: 10.1038/s41467-023-43411-y (PMC10665443; doi:10.1038/s41467-023-43411-y)
Supplement: Supplementary file 1 — Supplementary Information [file 41467_2023_43411_MOESM1_ESM.pdf]

Supplementary Information

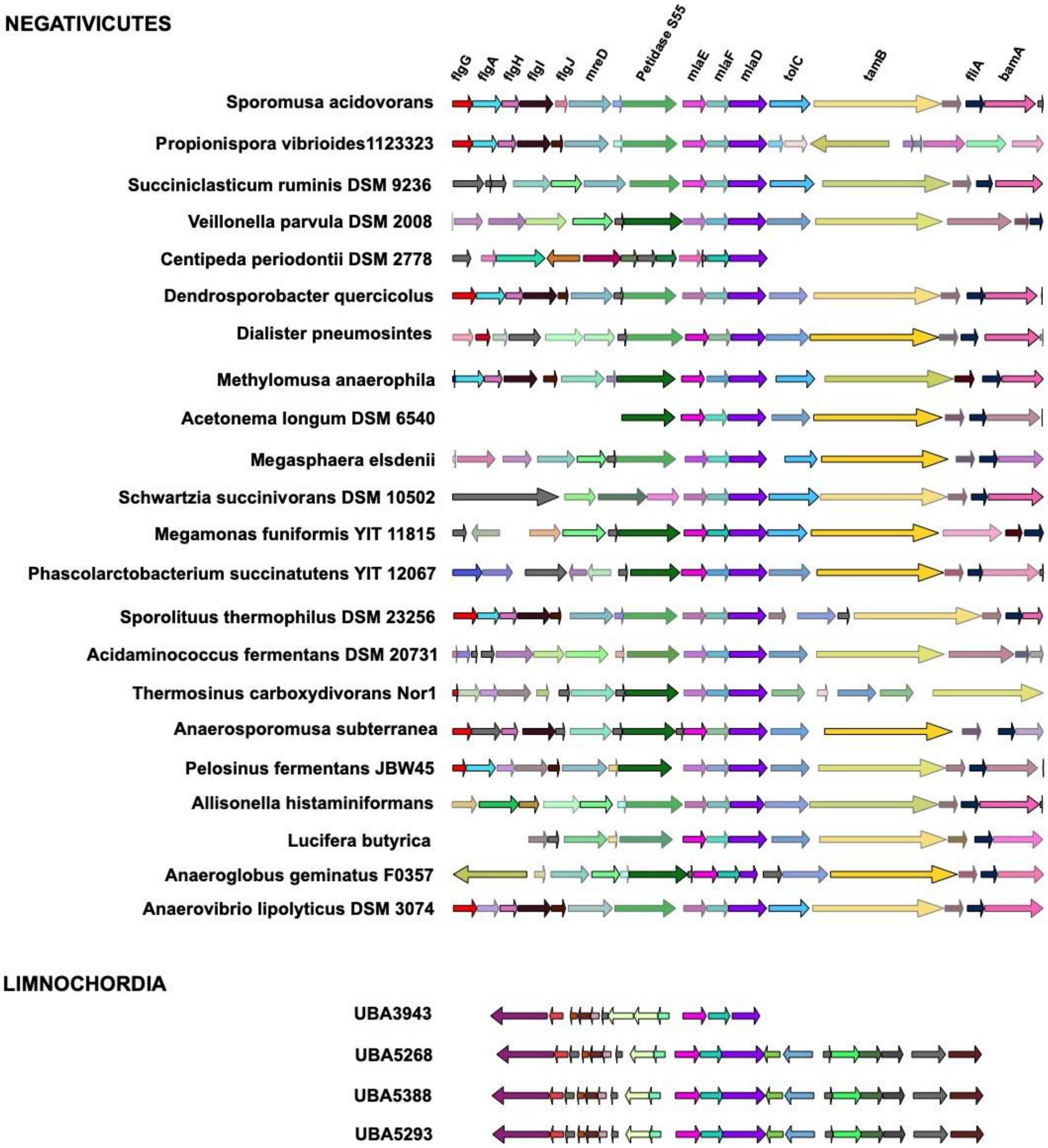

Supplementary Fig 1: Conserved synteny of *mla* genes with *tamB* and *tolC* in the Negativicutes, and presence of *mla* genes in Limnochordia

Within the Negativicutes, the three *mla* homologues, *mlaEFD*, are directly followed by a homologue of *tolC* and a homologue of *tamB*. This synteny is not conserved within the other diderm Firmicutes; no *mla* homologues were identified in the Halanaerobiales, whilst *mla* genes in the Limnochordia are spare and not in synteny with *tolC* and *tamB* homologues.

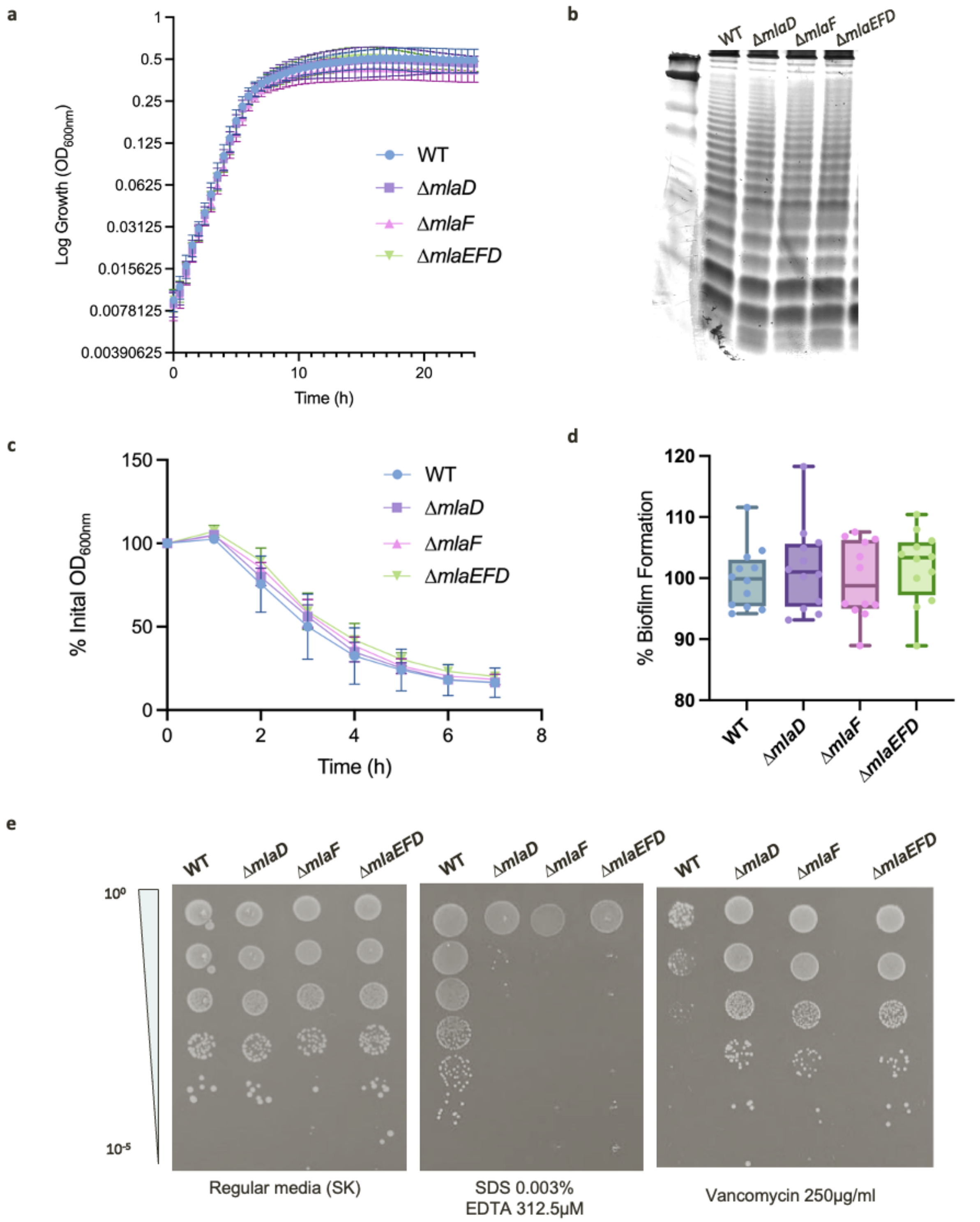

Supplementary Fig 2: Phenotypes resulting from deletion of *mla* genes.
a) Comparison of growth kinetics of WT and Δ*mla* mutants. All strains were assessed for growth kinetics via OD_{600} measurements via TECAN spectrophotometer (see methods). Δ*mlaD,* Δ*mlaF* and Δ*mlaEFD* were found to display similar growth rates to the WT, with no defects. At least 3 biological replicates and 3 technical replicates were performed per strain. **b) Comparison of LPS profiles.** LPS was extracted from WT and all Δ*mla* cultures, in triplicate, normalised by OD_{600}, then observed with silver staining. All strains display similar LPS profiles, in both banding pattern and quantity. **c) Comparison of autoaggregation capabilities.** OD_{600} was recorded for WT and Δ*mla* strains once an

hour for a period of seven hours. All strains display a similar decrease in OD_{600} over time, suggesting a similar capability of autoaggregation. At least 3 biological replicates and 3 technical replicates were performed per strain. **d) Comparison of biofilm formation.** Biofilm formation was assessed via crystal violet staining of biofilms formed over 24h in 96-well plates. Δ*mla* strains display a small but non-significant decrease in biofilm formation capability, as assessed by a decrease in fluorescence signal at A575nm. Due to variability in this technique, 10 biological replicates (n=10) with 3 technical replicates each were tested per strain. Significance calculated by two-tailed Mann Whitney U test. Box plot definition: the centre line denotes the median value (50th percentile), while the box contains the 25th to 75th percentiles of dataset. The whiskers mark the 5th and 95th percentiles. **e) Deletion of *mla* genes does not result in additive phenotype**. OM permeability of single and triple *mla* mutants were assessed via efficiency of plating on SDS 0.004% / EDTA 312.5µM and vancomycin (250µg/ml). Phenotypes of single *mla* mutants (Δ*mlaF* and Δ*mlaD*) are the same as those of the triple mutant (Δ*mlaEFD),* suggesting all three genes work together as part of one system.

a

V. parvula homologue	PL biosynthesis gene	Function
FNLLGLLA_00888	Phosphatidylserine decarboxylase (*psd)*	PE biosynthesis
FNLLGLLA_00889	Phosphatidylserine synthase (*pss),* phosphatidylglycerophosphate synthase (*pgsA*)	PE/PS biosynthesis<br>PG biosynthesis
FNLLGLLA_01855	Phosphatidylglycero-phosphatase (*pgp/ yutG)*	PG biosynthesis
FNLLGLLA_01719	*plsA/R*	Plasmalogen biosynthesis

b

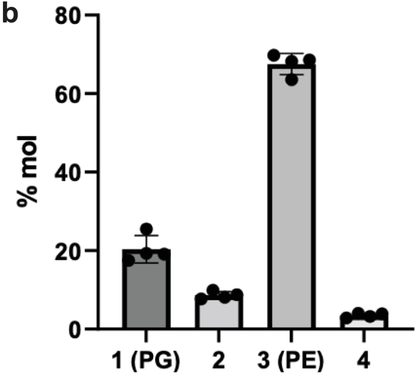

c

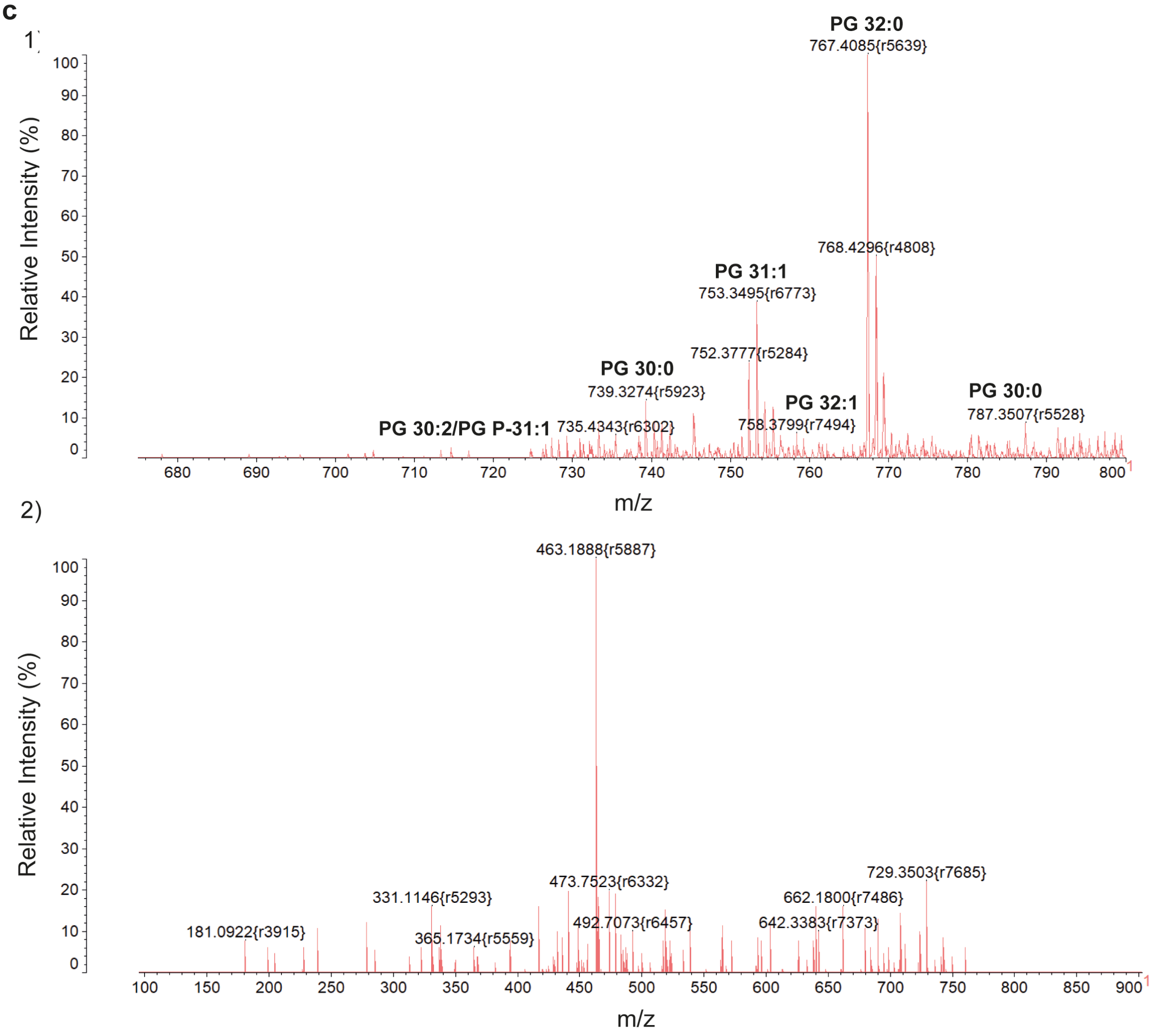

d

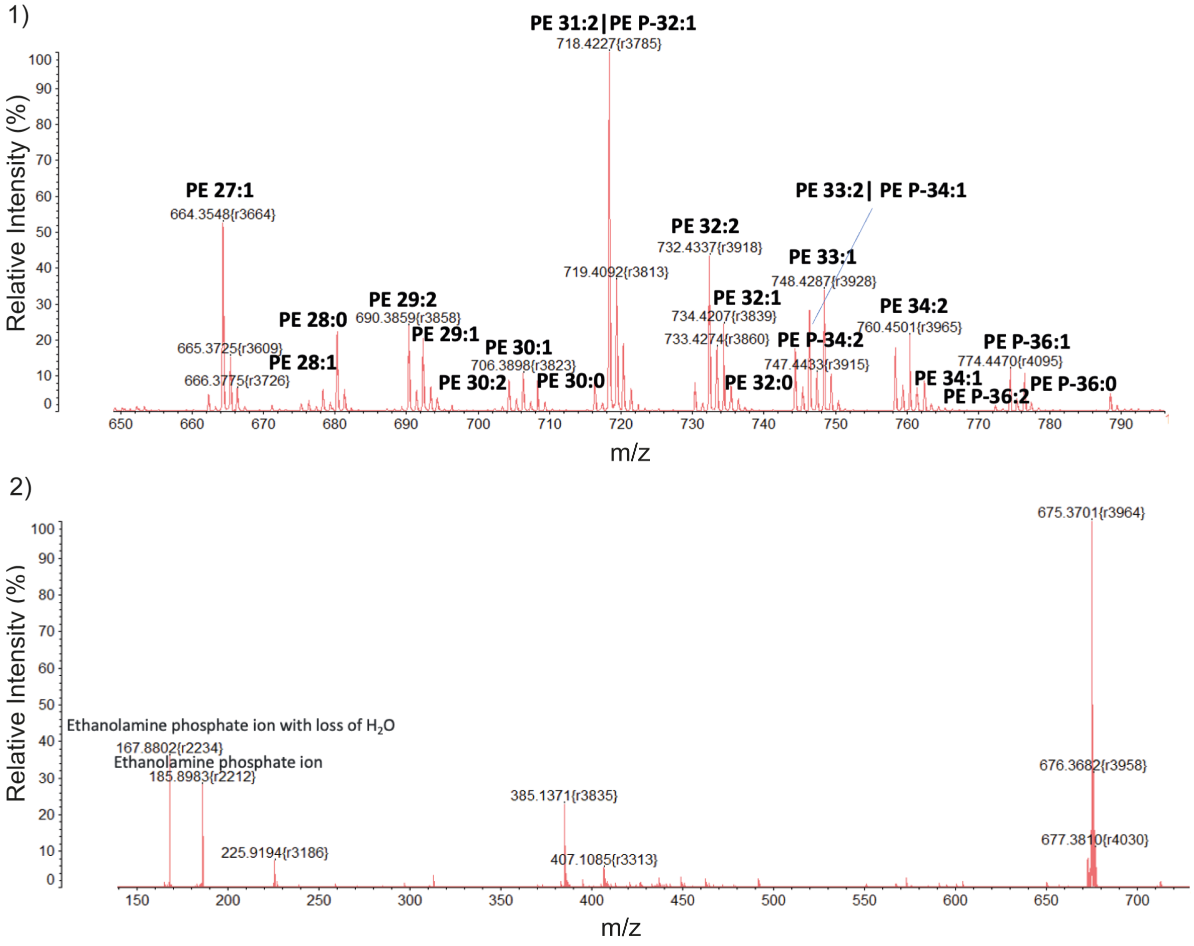

Supplementary Figure 3: GPL biosynthesis homologues in *V. parvula* and identification of PE and PG.

a) Identification of GPL biosynthesis homologues *in V. parvula*. Homologues of four GPL biosynthesis genes were identified in *V. parvula*, including a homologue for the recently identified operon involved in plasmalogen biosynthesis, *plsAR*. **b) Relative proportion of each lipid species in WT SKV38.** 4 biological replicates were tested, stained with phosphomolybdic acid and quantified by ImageJ; n = 4. **C) Mass spectrometry data – PG identification (band 1).** 1) MALDI-QIT-TOF MS analyses of lipid band 1 (positive ion mode) are shown to illustrate phosphatidylglycerol (PG) diversity from lipid extracts of WT *V. parvula* SKV38. 2) MS/MS spectrum of the main species of PG, PG 32:0 at *m/z* 767.408 with diagnostic ions for PG of 198.9 corresponding to the Glycerol-3-phosphate ion with loss of H_2O. Only one fatty acid neutral loss was found suggesting the presence of two C16:0. **d) Mass spectrometry data – PE identification (band 3).** 1) MALDI-QIT-TOF MS analyses of lipid band 3 (positive ion mode) are shown to illustrate phosphatidylethanolamine (PE) diversity from lipid extracts of WT *V. parvula* SKV38. 2) MS/MS spectrum of the main species observed by MS, PE 31:2 at *m/z* 718.4 with diagnostic ions for PE of 43 (neutral loss from the precursor ion) and 185 and 167 for respectively the ethanolamine phosphate ion and the ethanolamine phosphate ion with loss of H_2O.

Lipids	*m/z*	Assignment
Phosphatidylglycerol (PG)	735.434	PG 30:2 | PG P-31:1
	739.449	PG 30:0
	753.349	PG 31:0
	765.451	PG 32:1
	767.408	PG 32:0
	781.406	PG 33:0
Phosphatidylethanolamine (PE)	664.354	PE 27:1
	676.368	PE 28:2
	678.366	PE 28:1
	681.369	PE 28:0
	690.385	PE 29:2
	692.384	PE 29:1
	704.396	PE 30:2
	706.389	PE 30:1
	708.387	PE 30:0
	718.422	PE 31:2|PE P-32:1
	732.433	PE 32:2
	734.420	PE 32:1
	736.425	PE 32:0
	744.423	PE P-34:2
	746.440	PE 33:2| PE P-34:1
	748.428	PE 33:1
	760.450	PE 34:2
	762.442	PE 34:1
	772.470	PE P-36-2
	774.447	PE P-36:1
	776.457	PE P-36:0

Supplementary Table 1. Lipid assignments of the total lipid extract of *Veillonella parvula.* Analyses by MALDI-QIT-TOF Shimadzu AXIMA Resonance mass spectrometer in the positive mode. The adduct type for phosphoethanolamine (PE) and phosphatidylglycerol (PG) are [M+2Na-H]+.

a

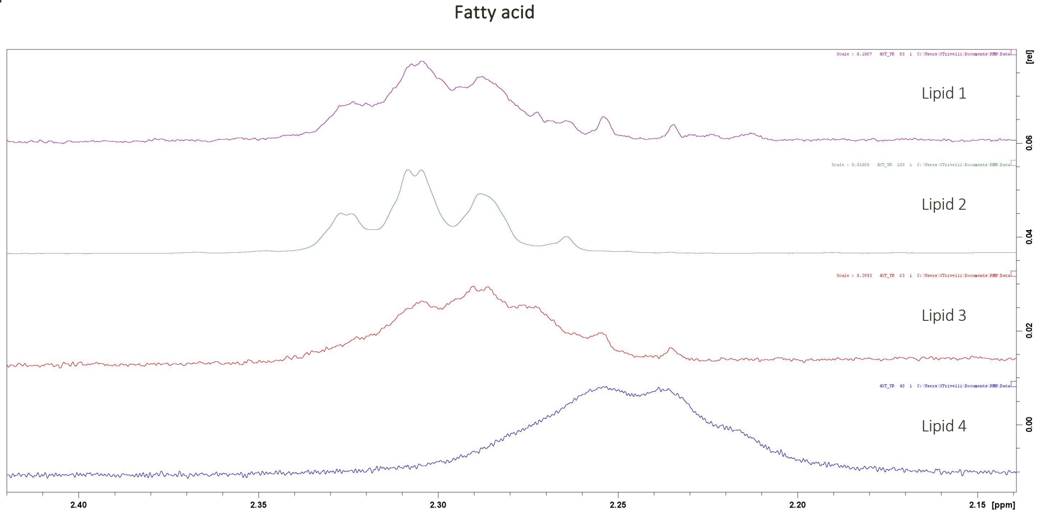

b

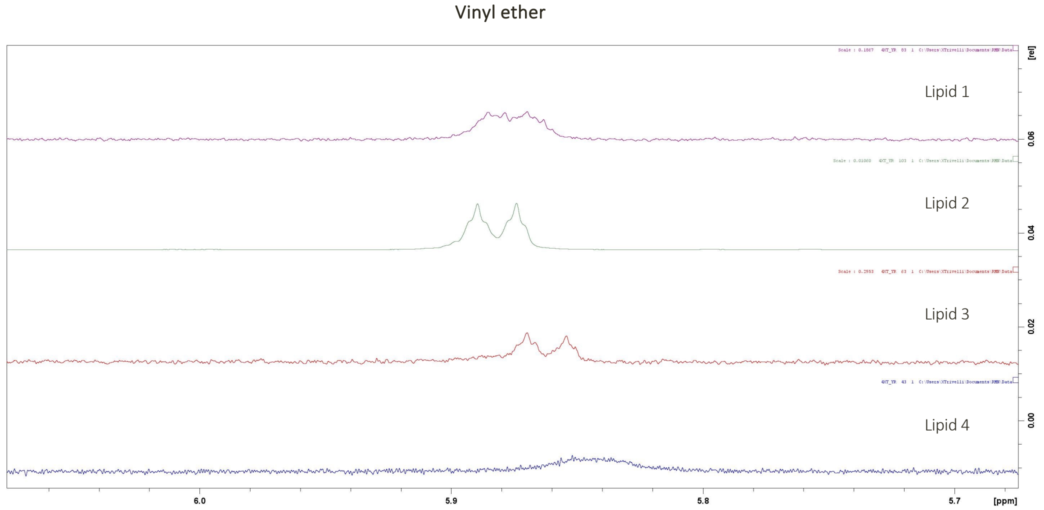

c

Band number	Lipid Identity	Ratio of vinyl ether (VE) to ester (E) fatty acid bonds	Relative % of vinyl ether lipid	Number of independent measures
1	PG	1.0 VE 6.2 E	14	3
2	Unknown	1.0 VE 5.6 E	15	2
3	PE	1.0 VE 1.8 E	36	2
4	Unknown	1.0 VE 3.5	22	5

Supplementary Figure 4: Identification of vinyl ether bonds in *V. parvula* phospholipids
a) Proton Nuclear Magnetic Resonance (^{1}H-NMR) spectra of lipid fractions 1-4 used for the relative quantification of Ester moieties (FA). Relative quantification was made using signal area from the alpha-methylene of the fatty acids (FA) (two ^{1}H signals at ca. 2.30 ppm per FA). **b)** ^{1}H-NMR spectra for relative quantification of Vinyl Ether (VE) bonds. Relative quantification was made using signal area for the VE moiety (one ^{1}H at 5.88 ppm per VE chain). **c)** Relative proportion of plasmalogen form of each lipid. Based on the relative molar quantity of each of the four major lipids present in the WT, and the NMR data, we calculated the relative molar proportion of lipids containing ether bonds.

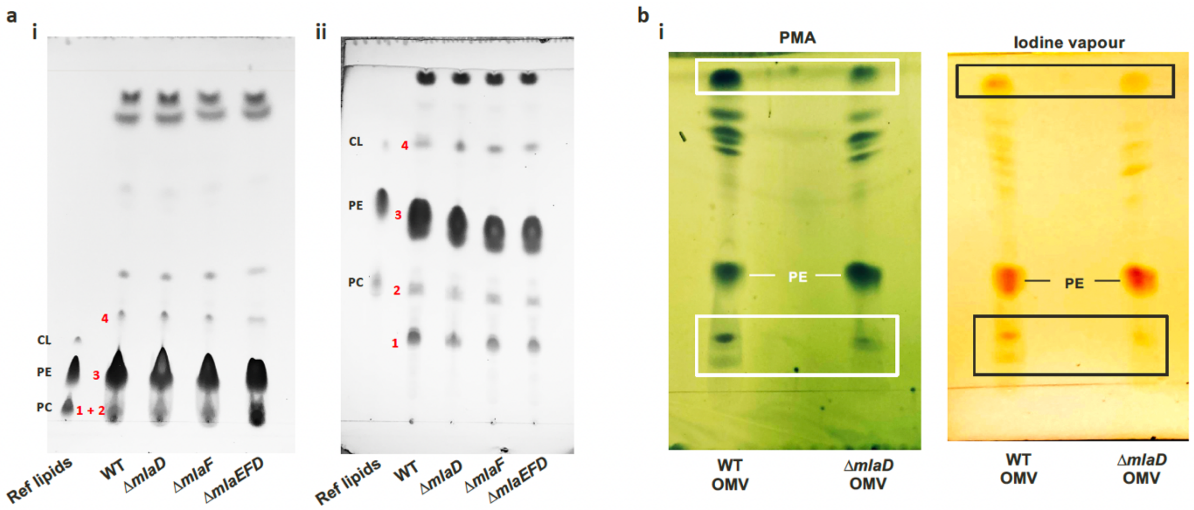

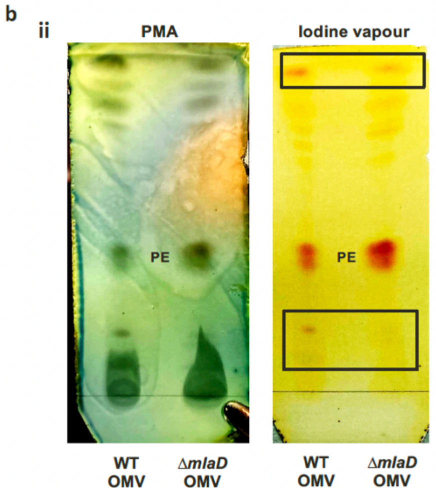

	WT PE	ΔmlaD PE	Difference	% increase
Bio rep 1	521092	632613	111521	21.4
Bio rep 2	316973	478901	161928	51.1
Bio rep 3	165687	242523	76836	46.4
Average				39.6
STD				16.0

Supplementary Figure 5: Phospholipid compositions of WT and Δ*mla* *V. parvula* mutants from whole-cell lipid extracts and purified outer membrane vesicles (OMVs)

a) Thin Layer Chromatography (TLC) of whole-cell extracted lipids with iodine vapour staining. **i)** Solvent system was chloroform/methanol/water (v/v/v 80:20:2.5). A strong clustering of polar species was observed near the origin of the plate. **ii)** Solvent system was chloroform/methanol/water (v/v/v 65:25:4). This solvent system enabled a better separation of the more polar species, allowing the separation of PE as a double band, likely representative of two different PE species. The major lipid species in *V. parvula*, listed in the table below, are numbered 1 – 4. Commercial reference lipid standards (PC, PE and CL) are labelled. **b) i) TLC of lipids extracts from OMVs: Phosphomolybdic acid (PMA) and iodine vapour staining** (chloroform/methanol/water 80:20:2.5 v/v/v)**.** Lipid extraction was performed from purified OMVs of WT and Δ*mlaD* strains in biological triplicate as described in the methods. 10µl of extracted lipids were run on TLC, revealing a relative enrichment of PE in all Δ*mlaD* OMV lipid extracts as compared to lipid 2. PMA staining of lipid extracts from WT and Δ*mla* OMVs shows a decrease in the relative quantities of two unknown lipid species, highlighted in white, as opposed to enhanced detection of PE. This decrease was also visualised after iodine vapour staining, as highlighted in black. **b) ii)** Additional biological replicate of OMV lipid extraction and staining, including a table showing ImageJ quantifications of staining intensity of iodine vapour-stained lipid extracts across 3 biological replicates.

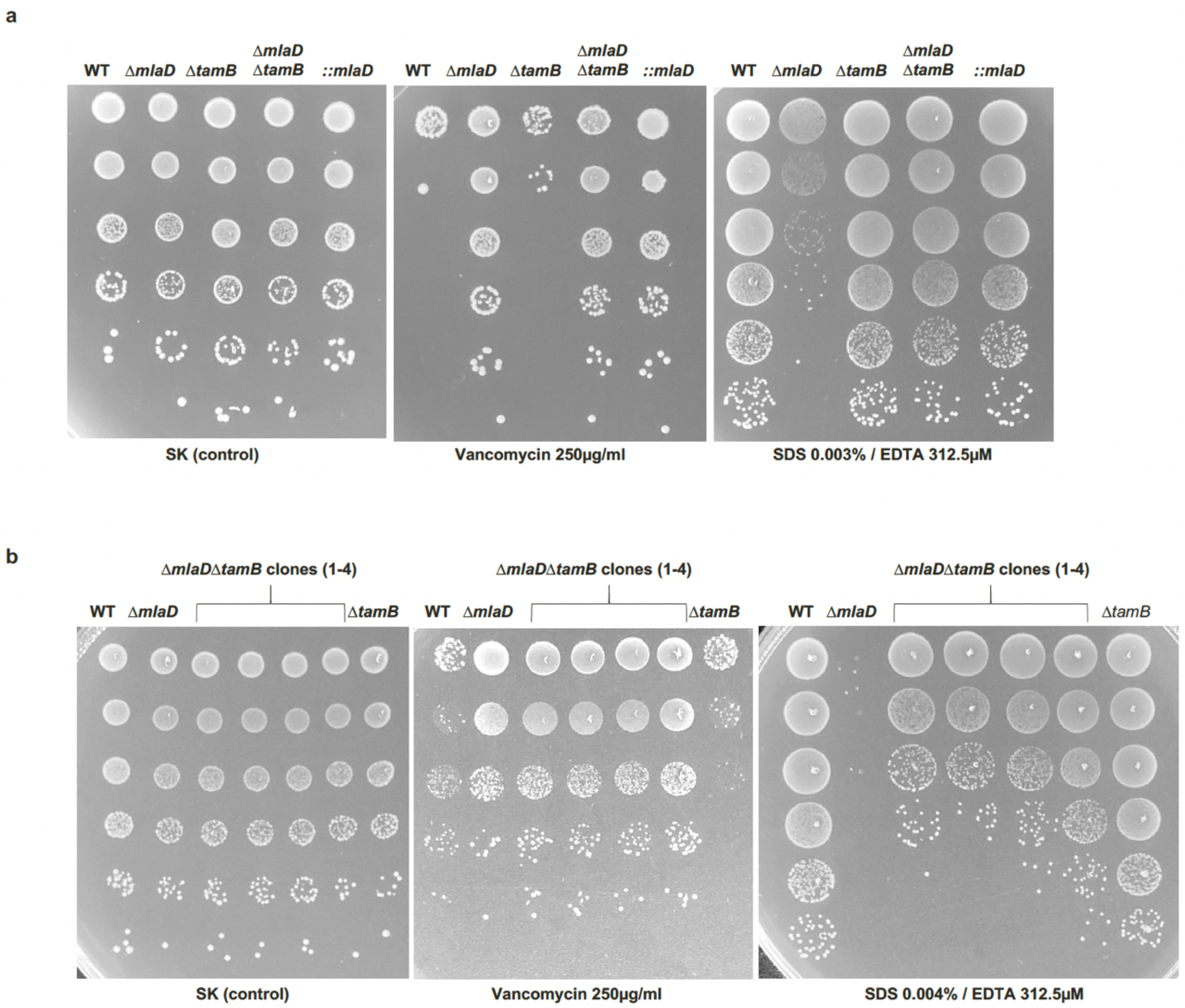

Supplementary Figure 6: OM permeability phenotypes of Δ*mlaD*, Δ*tamB* and Δ*mlaD*Δ*tamB* strains

a) Overnight cultures of WT, Δ*mlaD,* Δ*tamB,* Δ*mlaD*Δ*tamB* and Δ*mlaD::mlaD* were plated onto SDS / EDTA and vancomycin to assess OM permeability. At this concentration of SDS (0.003%) and EDTA (312.5µM), the rescue of detergent hypersensitivity of Δ*mlaD* by deletion of *tamB* is striking, and similar to the rescue obtained from reintroducing the expression of *mlaD* itself. Δ*tamB* strains are also highly sensitive to vancomycin. **b)** Cultures of WT, Δ*mlaD,* Δ*tamB* and 4 independent biological replicates of the double Δ*mlaD*Δ*tamB* mutant were plated onto SDS 0.004% / EDTA 312.5µM and vancomycin 250µg/ml. The slight increase in vancomycin sensitivity of the Δ*tamB* can be observed as compared to the WT, which is in direct contrast to the high resistance of Δ*mlaD* to vancomycin.

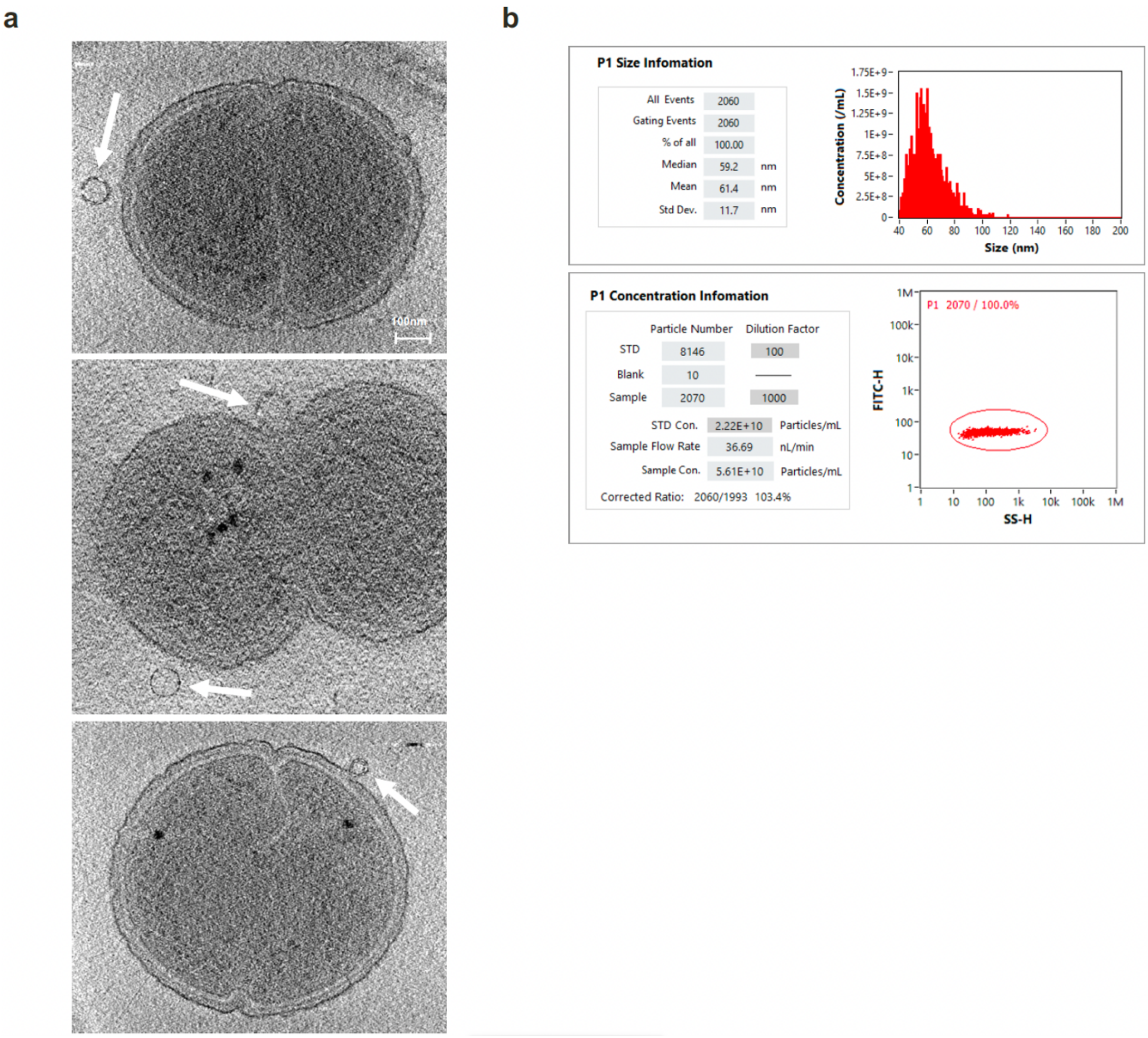

Supplementary Figure 7: Average size of outer membrane vesicles (OMVs) is consistent across whole cell cultures and purified OMV samples.

a) Cryo-electron tomography images of Δ*mlaD* with outer membrane vesicles (OMVs). Cultures of WT and Δ*mlaD* strains were observed via cryo-electron tomography (cryo-ET). In all tomograms of Δ*mlaD*, outer membrane vesicles (OMVs) were observed either attached to the cell or externally (highlighted by white arrows). These vesicles were on average ~60nm in diameter, matching the average size observed of these vesicles (present both in whole-cell cultures and in purified samples) via NanoFCM. Scale bar = 100nm. **b) NanoFCM report showing size distribution of purified OMV sample.** OMVs were purified from large-scale supernatants of WT and Δ*mlaD* cultures. Prior to concentration of these vesicles via ultracentrifugation, a small sample was extracted and processed via NanoFCM, confirming the average size of OMVs is ~60nm, matching the average size of OMVs present in full cell cultures of WT and Δ*mlaD* strains, and also the average size observed via cryo-ET.

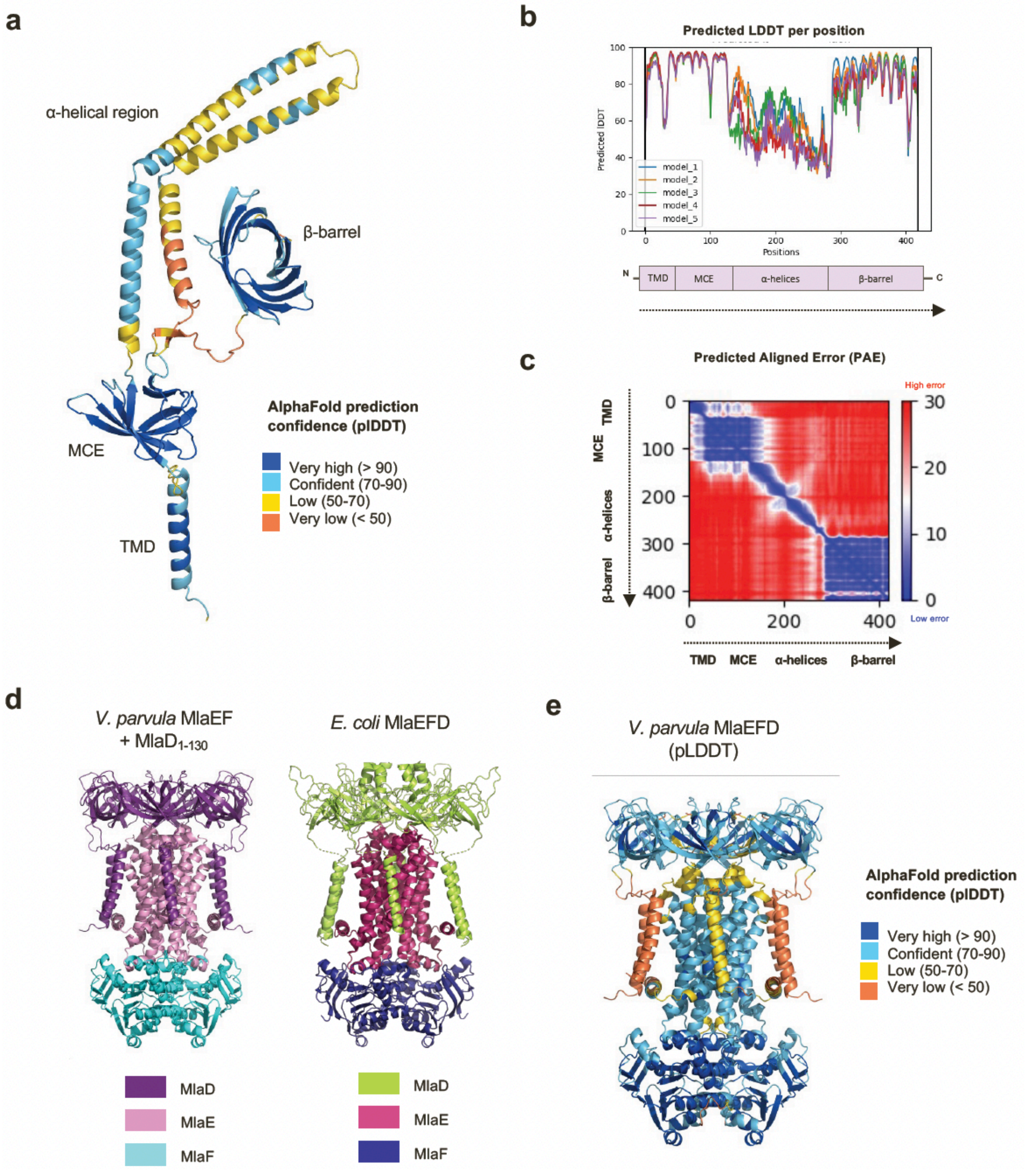

Supplementary Figure 8: AlphaFold2 modelling of MlaD in *V. parvula* **a) Predicted structure of full-length MlaD.** When the whole sequence is processed by AlphaFold2, the TMD, MCE and β-barrel domains are of very high confidence, and the α-helical domain is of lower confidence. The confidence rating is indicated by Predicted Local Distance Different Test (pLDDT) colouring. **b) Predicted Local Distance Difference Test (pLDDT) per position of full-length MlaD.** Graphical depiction of the pLDDT per position of the full-length models shows low confidence for the α-helical region, and high confidence for the TMD, MCE and β-barrel domains. **c) Predicted Aligned Error (PAE) of full-length MlaD.** Graphical depiction of the PAE shows the confidence in the relative positioning of the domains. The relative positioning of residues within the TMD and MCE domains and within the connecting α-helical region are low error, suggesting the predicted local conformation of these individual domains to be likely. However, the high predicted error of residue pairs across these domain regions indicates that the global conformation of full-length MlaD is likely inaccurate. **d) Modelling of MlaEFD complex in *V. parvula* with a 2:2:6 stoichiometry.** MlaEF modelled as dimers in complex with hexameric MlaD (residues 1-130) closely resemble the resolved structure of the MlaEFD complex in *E. coli.* The AlphaFold model of $MlaEFD_{1\text{-}130}$ can be found in the Supplementary Dataset 2. **e)** pLDDT per position of MlaEFD in *V. parvula* shows high confidence with the exception of 2 pairs of TM helices.

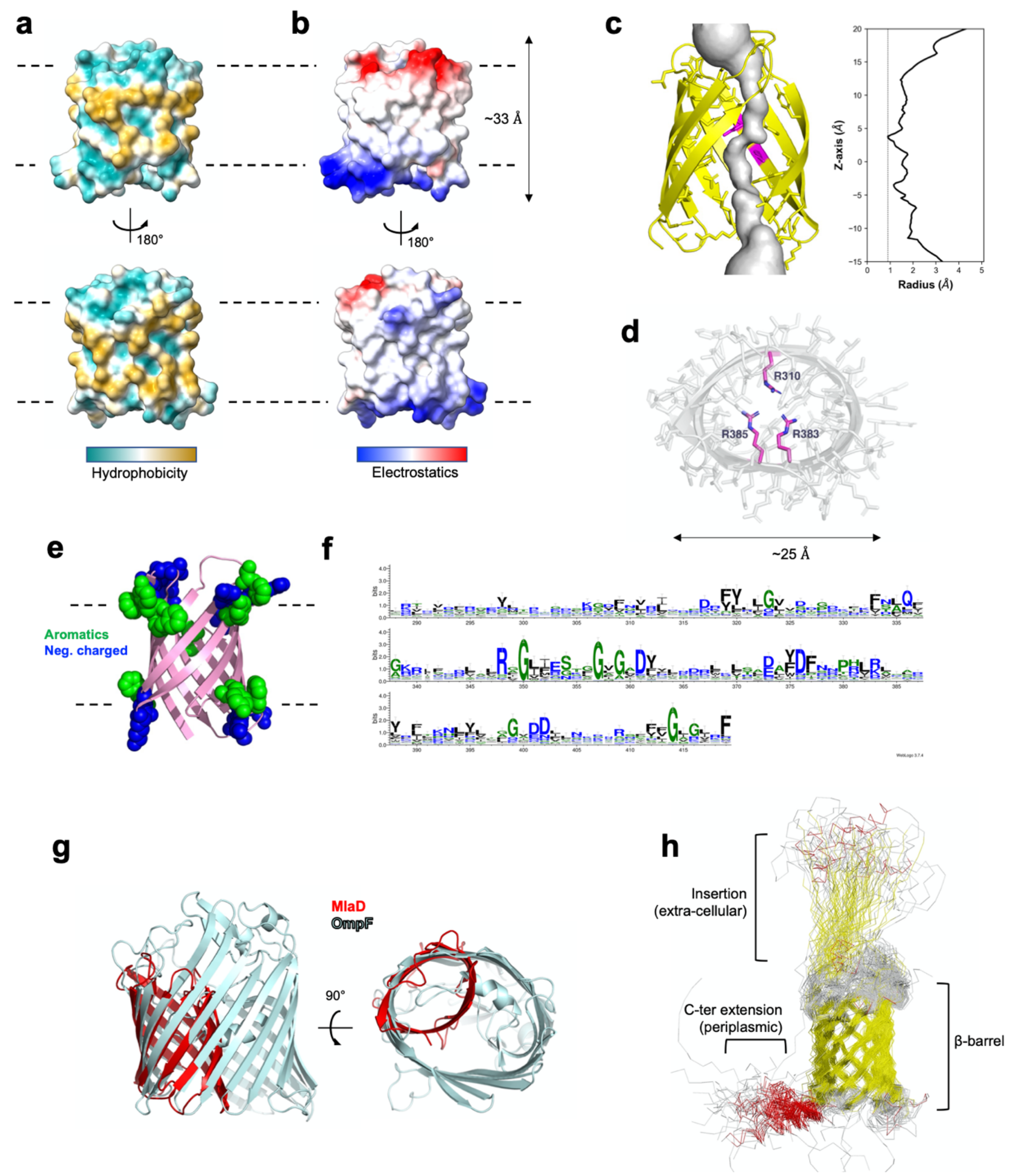

Supplementary Fig 9: The AlphaFold predicted C-terminal domain of MlaD ($MlaD_{288-419}$) folds as a membrane β-barrel.

a) Hydrophobicity potential of the predicted MlaD C-terminal β-barrel surface. The bottom view is rotated 180°. Dotted lines represent the possible position of GPL polar heads when inserted in a lipid bilayer. **b) Electrostatics potential of the predicted MlaD C-terminal β-barrel surface**. The bottom view is rotated 180°. The height of the barrel matches the thickness of a lipid bilayer**. c)** Pore channel in the core of the β-barrel and pore radius profile (calculated using the program HOLE[4]) along the Z-axis (membrane normal). Side-chains of R383 and R385 are colored in magenta. For sake of clarity, the first two β-stands are not shown. **d)** Position of side-chains of R310, R383 and R385 at the constriction point of the β-barrel. The width of the barrel is indicated. **e)** Rings of surface accessible negatively charged and aromatics residues at each side of the β-barrel. **f)** Weblogo of MlaD C-terminal β-barrel sequences colored by hydrophobicity (Hydrophilic in blue, neutral in green and hydrophobic in black). **g)** Superimposition of the *V. parvula* MlaD C-terminal β-barrel model (red) onto the *S. marcescens* OmpF structure (light cyan). Its small diameter, compared to large porins such as OmpF,

and very narrow inner radius (~1 Å) would likely prevent the passage of any small molecule. **h)** Superimposed ensembles of MlaD C-terminal β-barrel models for long MalD sequences predicted with AlphaFold2. Helices are colored in red and β-strands in yellow. Insertions in the barrel and C-terminal helical extensions found in some sequences are labeled.

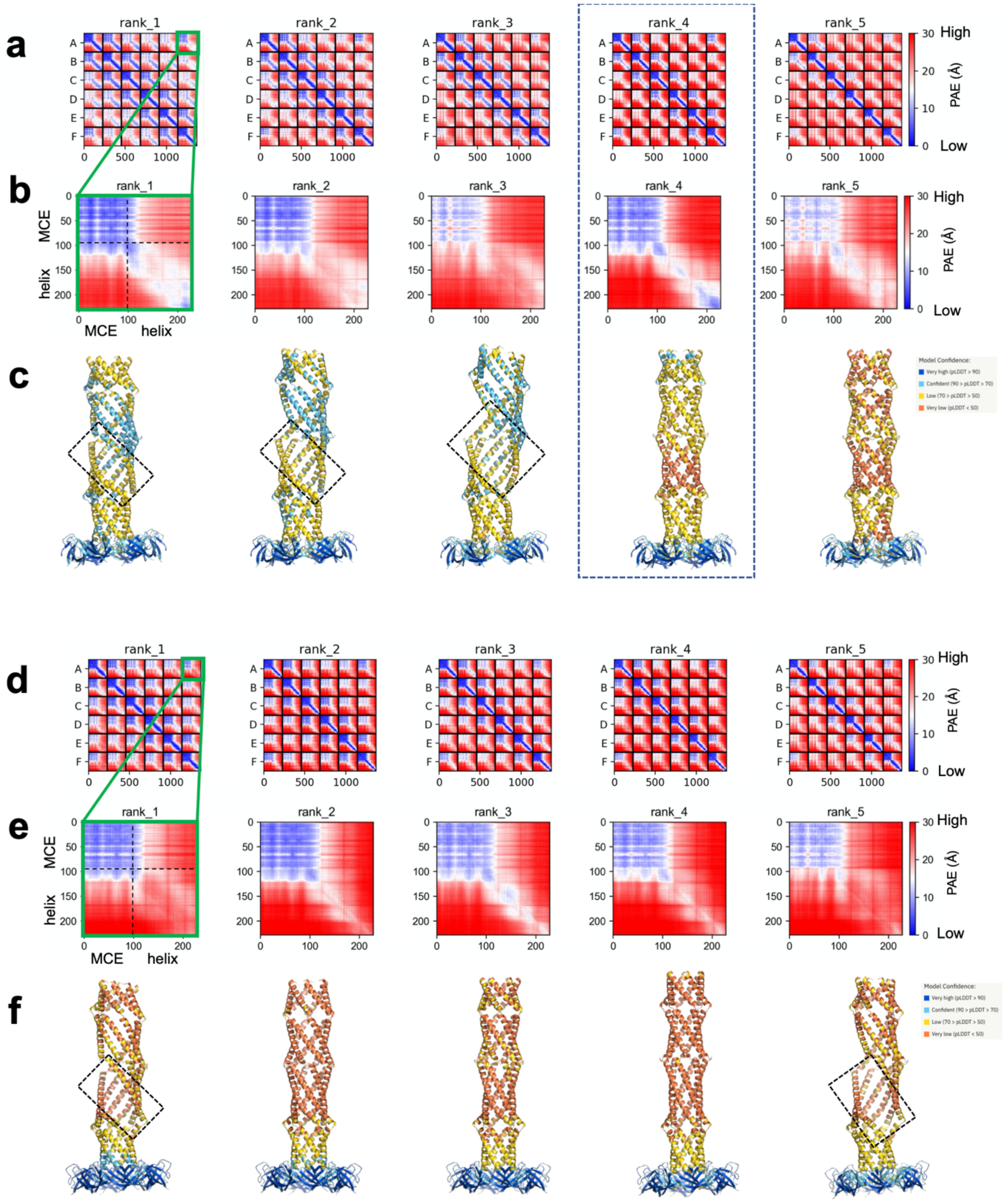

Supplementary Fig 10: AlphaFold2 models for the alpha-helical domain of MlaD$_{36\text{-}263}$ in hexameric configuration.

a) Predicted Aligned error (PAE) for the 5 ranked models obtained with AlphaFold-Multimer v2.2. The regions corresponding to each chain in the hexamer (from A to F) are labeled. **b)** PAE between the first (A) and last (F) chain for the 5 ranked models obtained with AlphaFold-Multimer v2.2. The regions corresponding the MCE and helical domains are labeled. **c)** Models obtained AlphaFold- Multimer v2.2. colored by predicted local distance difference test (pLDDT). Black dashed boxes indicate the position of an "open groove" when the first (A) and last (F) helices are not in contact. The blue dashed box indicate the AF-multimer model of MlaD$_{36\text{-}263}$ that was used to build the MlaD$_{Vp}$ full-length model. **d)** Predicted Aligned error (PAE) for the 5 ranked models obtained with AlphaFold-Multimer v2.3. The regions corresponding to each chain in the hexamer (from A to F) are labeled. **e)** PAE between the first

(A) and last (F) chain for the 5 ranked models obtained with AlphaFold-Multimer v2.3. The regions corresponding the MCE and helical domains are labeled. **f)** Models obtained AlphaFold-Multimer v2.3 colored by predicted local distance difference test (pLDDT). Black dashed boxes indicate the position of an “open groove” when the first (A) and last (F) helices are not in contact.

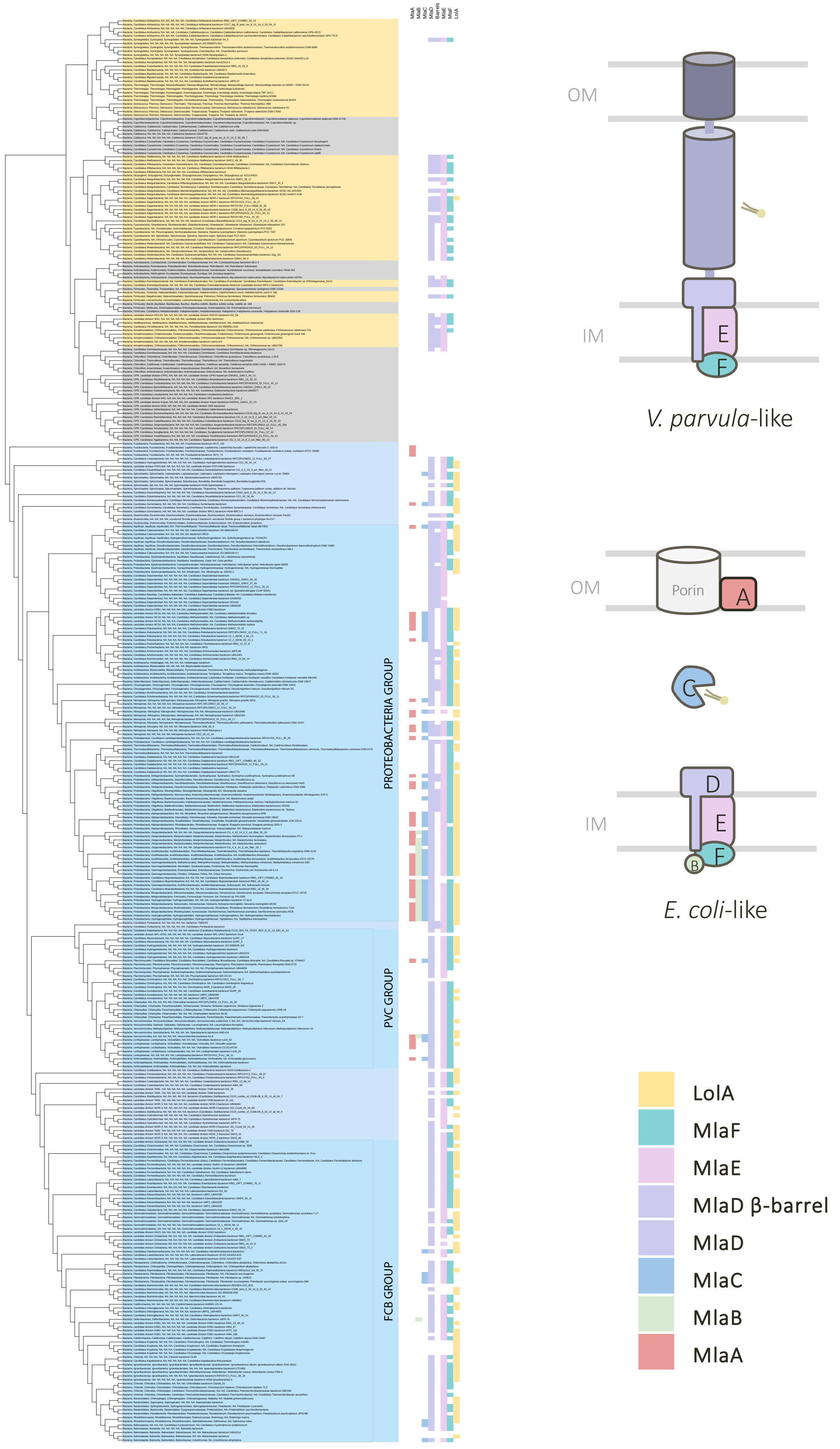
PROTEOBACTERIA GROUP
PVC GROUP
FCB GROUP
OM
IM
E
F
V. parvula-like
OM
Porin
A
C
IM
D
E
F
B
E. coli-like
LolA
MlaF
MlaE
MlaD β-barrel
MlaD
MlaC
MlaB
MlaA

Supplementary Fig 11: Taxonomic distribution of Mla components (and LolA) with species names

This figure is adapted from main text Fig 5 to include detailed species names. All other aspects of the figure are the same. Presence or absence of Mla component indicated in red (MlaA), green (MlaB), blue (MlaC), purple (MlaD), pink (MlaE), turquoise (MlaF) and LolA is indicated in yellow. (Yellow / Grey = Terrabacteria; Blue = Gracilicutes). Tree generated using custom made scripts and iTOL[5].

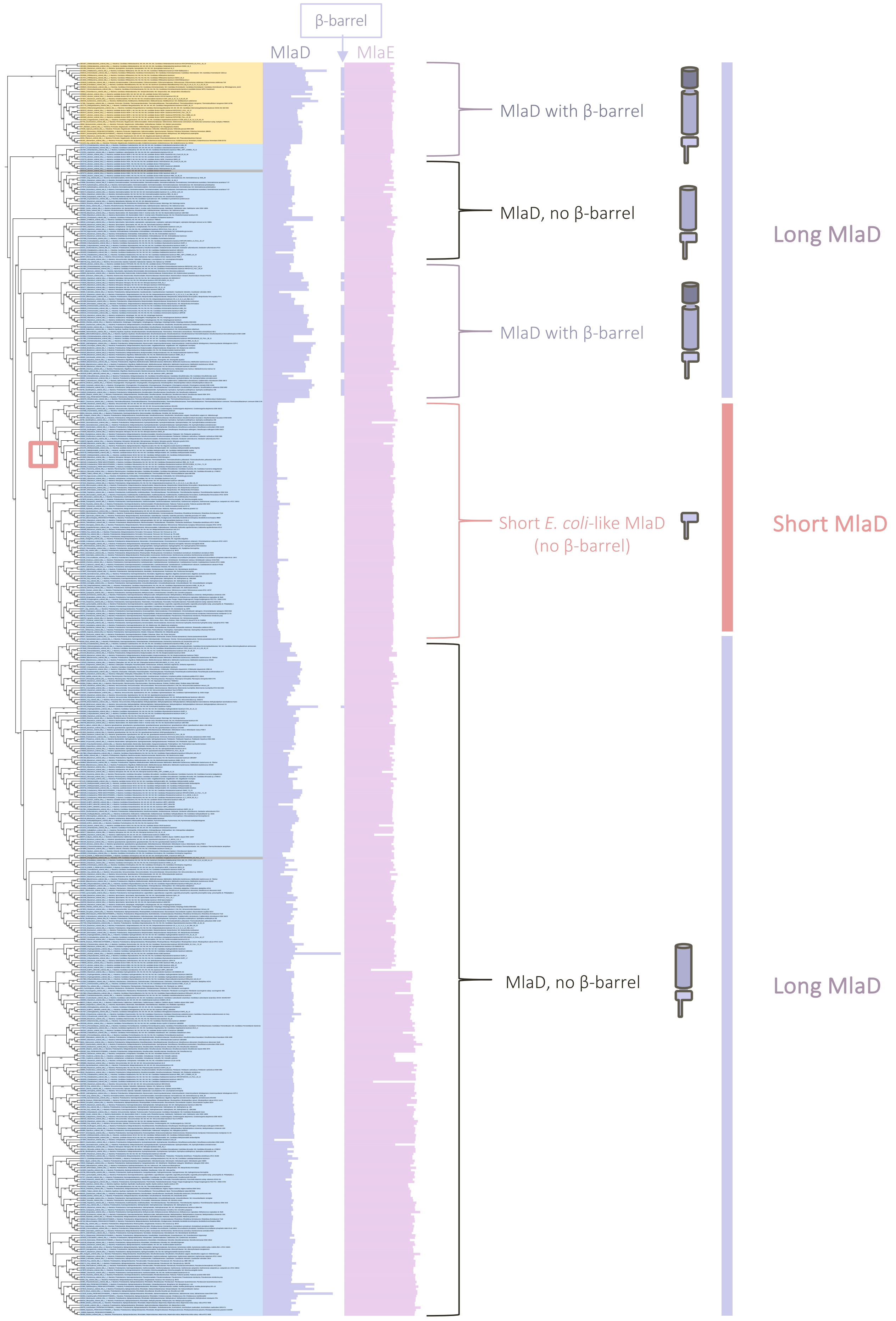

β-barrel
MlaD
MlaE
MlaD with β-barrel
MlaD, no β-barrel
MlaD with β-barrel
Short *E. coli*-like MlaD
(no β-barrel)
MlaD, no β-barrel
Long MlaD
Short MlaD
Long MlaD

Supplementary Fig 12: Phylogeny concatenation of MlaE with the three types of MlaD: short, long and long with β-barrel

Sequences of MlaE and MlaD were concatenated into a character supermatrix (538 sequences and 479 amino acid positions) and a maximum likelihood tree was inferred. The length of MlaD sequence is indicated by the length of the purple bars, and the presence / absence of the β-barrel is depicted with a small purple at the end of the sequence. MlaE sequences are represented in pink. We clearly see from this analysis that the majority of MlaD sequences are long, and that the short MlaD sequences are restricted mostly to the Proteobacteria. We also see a clear separation between the Terrabacteria (yellow) and the Gracilicutes (blue), with monophyly of phyla within the Terrabacteria, indicating vertical inheritance of these Mla genes. Highlighted in the red box is the branch at which loss of this elongated form of MlaD seems to have occurred.

Tree scale: 1

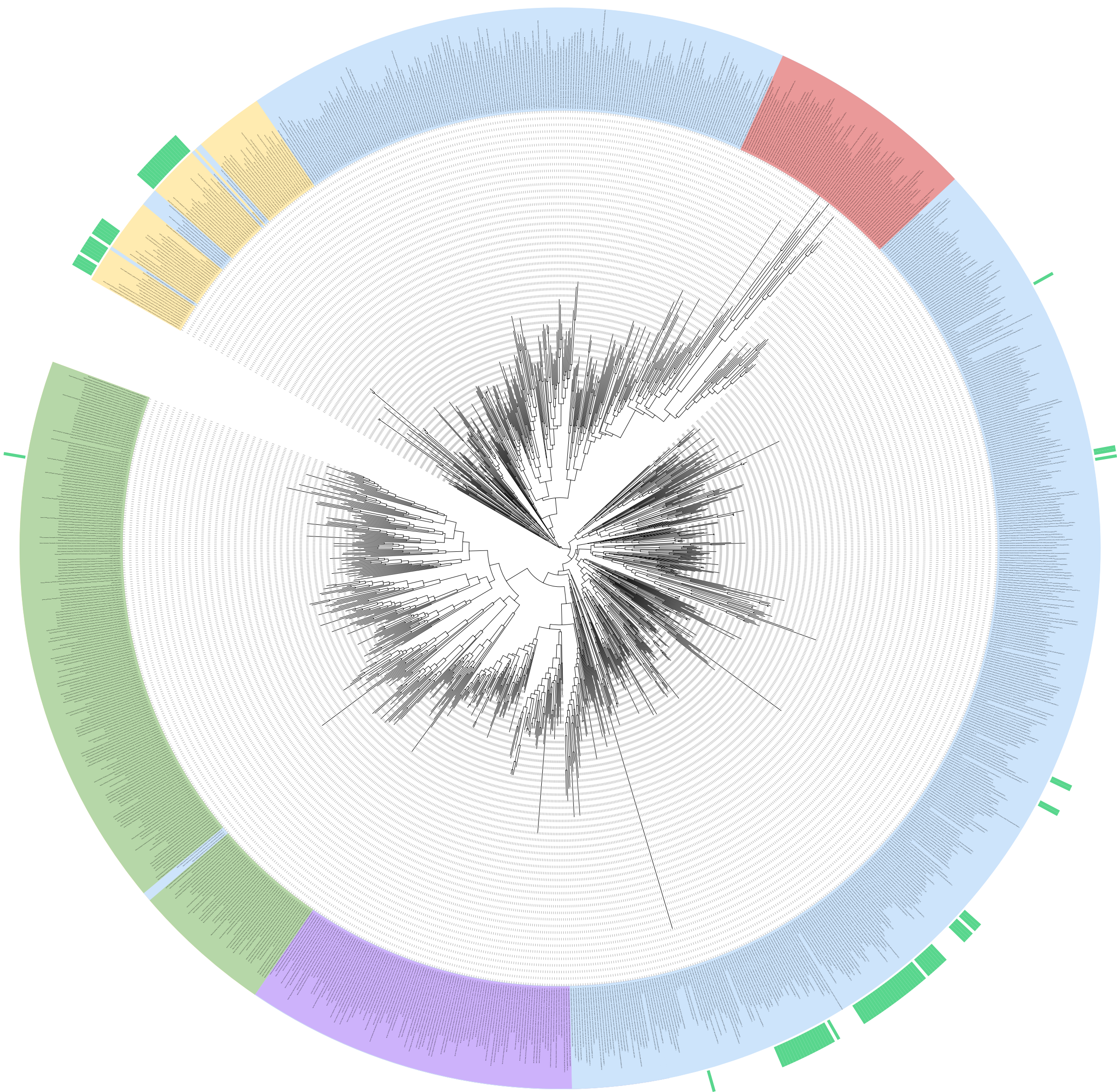

Supplementary Fig 13: Distribution of MCE-containing sequences across Bacteria

Maximum likelihood tree built from an alignment of 1119 sequences and 194 amino acid positions. The scale bar corresponds to the average number of substitutions per site. MlaD sequences are marked in green for Actinobacteria and C. Dormibacteraeota, yellow for other Terrabacteria and blue for Gracilicutes. We highlighted the short version of MlaD in purple, and PqiB and LetB sequences in red. The presence of a β-barrel is highlighted by a green bar at the outer ring of the tree.

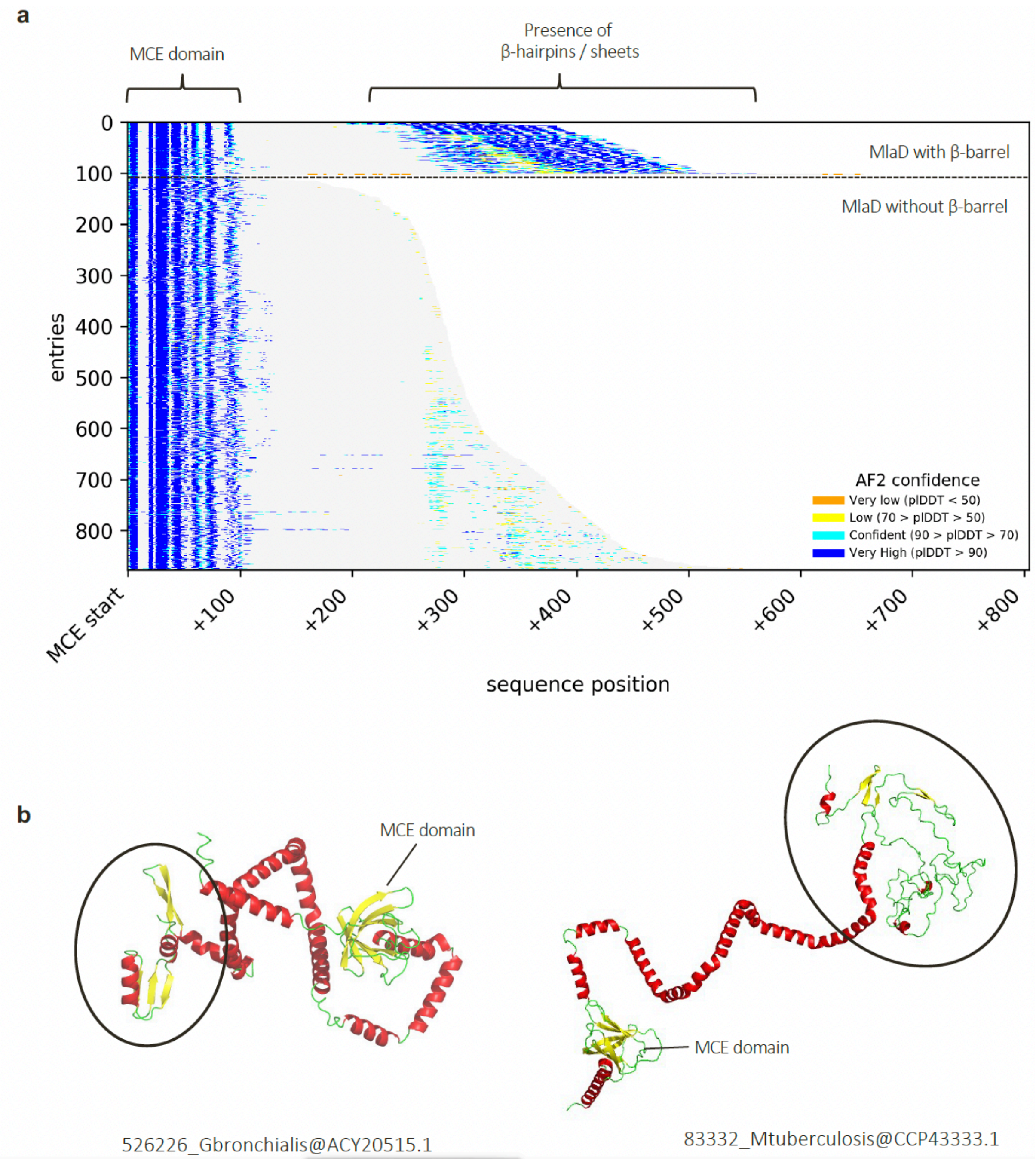

Supplementary Fig 14: Presence of disordered β-structures in long MlaD sequences with no β-barrel

a) Presence of β-structures in long MlaD sequences with no β-barrel. The start of the sequence represents the presence of the MCE domain, to align all long MlaD sequences. The total length of each sequence is represented by the grey shaded area. Later, in some of these long sequences, we see the presence of β-hairpins / sheets, but no real folded β-barrel domain. For comparison, we also show the ~100 sequences of long MlaD that do possess a predicted folded β-barrel. Predicted Local Distance Difference Test (pLDDT) values, corresponding to the confidence of these predictions, are coloured and labelled in the key above. **b) AlphaFold modelling of long MlaD sequences with no β-barrel.** As seen from these two predicted structures, these long MlaD sequences do not possess a β-barrel, but do possess unstructured, disordered regions at their C-termini that contain fragments of β-hairpins / sheets.

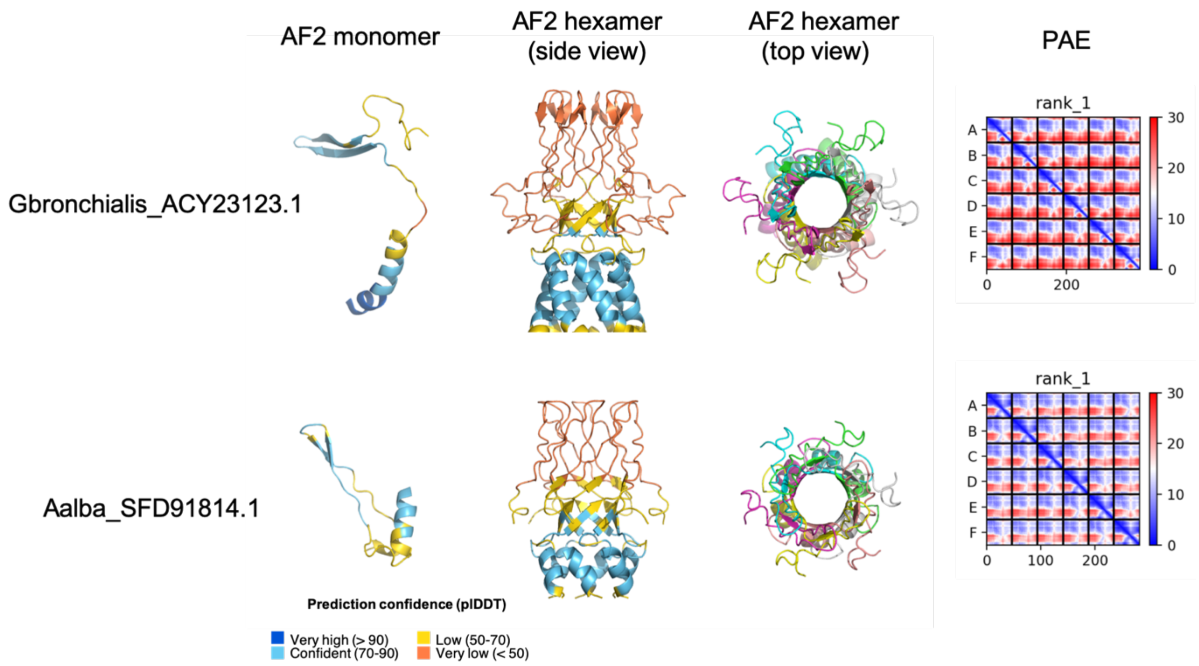

Supplementary Figure 15: Hexameric models of C-terminal domains from two representative MlaD homologues without a C-terminal β-barrel

Predicted models for C-terminal domains of 526226_Gbronchialis@ACY23123.1 and 673379_Aalba@SFD91814.1 as monomers or hexamers are presented. **From left to right**: i) Cartoon representation of the C-terminal domain structure predicted as monomer by AlphaFold2 and colored by plDDT values per residue from orange (low) to high (dark blue); ii) Cartoon representation of the predicted homo-hexameric assemblies of the same C-terminal domains in side view colored by plDDT values per residue; iii) Top view colored by subunits; iv) Predicted Aligned Error (PAE) maps of the homo-hexameric models, colored from blue (low) to red (high) error. When predicted as a monomer, the C-terminal domains display α-helical and β-strand content but without proper three-dimensional folding. When predicted as hexamers, they assemble to form a small β-barrel where each subunit contributes with a single strand. The low PAE values between subunits (off-diagonal squares) indicate that the hexameric assembly is confidently predicted by AlphaFold-Multimer.

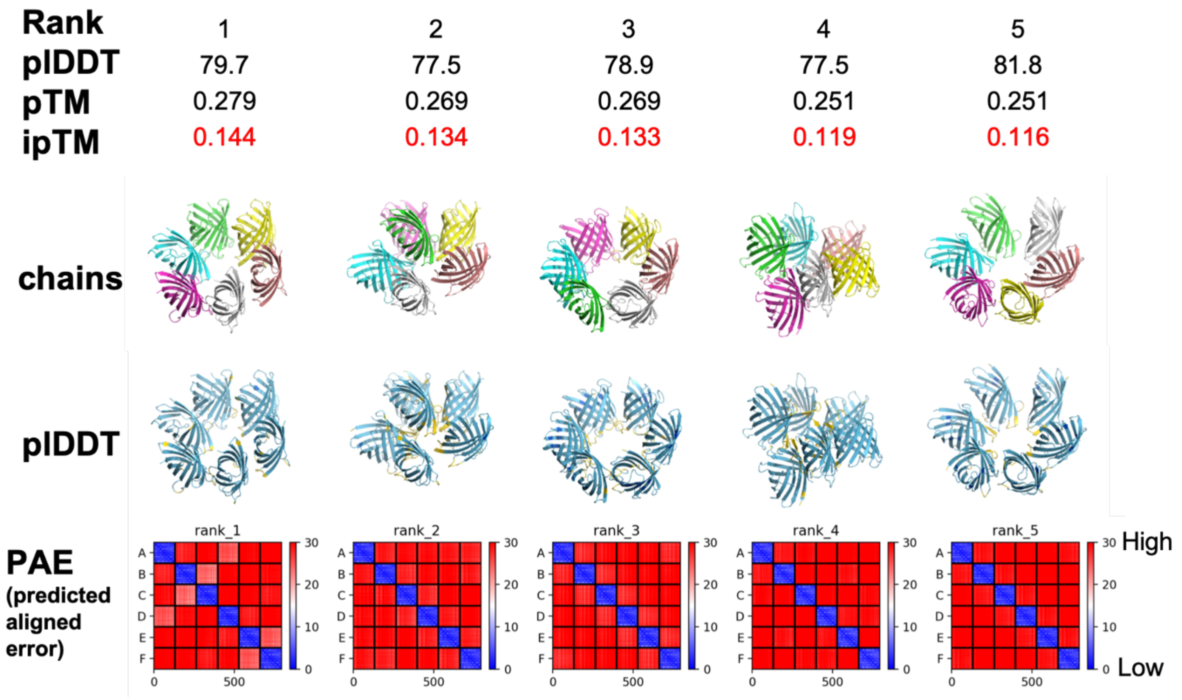

Supplementary Figure 16: Hexameric models of *Vp* MlaD$_{286\text{-}419}$

The statistics and representations of the 5 models predicted by AlphaFold-Multimer predictions for hexamers of the MlaD C-terminal β-barrel are presented. **(Top)** predicted Local Distance Difference Test (plDDT), predicted TM-score (pTM) and interface predicted TM-score (ipTM) values for the 5 ranked models. **(Middle)** Cartoon representation of the models colored by subunits (chains) or by plDDT values per residue from orange (low) to high (dark blue). **(Bottom)** The corresponding Predicted Aligned Error (PAE) maps. The low ipTM values as well as the very high PAE values between subunits (off-diagonal squares) indicate that the models are of poor confidence with regards to the relative position and orientation of the subunits.

Supplementary Table 2: *Statistics of AF2-Multimer models generated for MlaD36-263 hexamers*

	AF-multimer v2.2					AF -multimer v2.3				
Model rank	1	2	3	4	5	1	2	3	4	5
Open/Closed	Open	Open	Open	**Closed**	**Closed**	Open	**Closed**	**Closed**	**Closed**	Open
pLDDT	75.38	75.57	73.79	68.07	62.59	68.23	64.88	65.96	64.24	64.73
pTM	0.56	0.55	0.50	0.47	0.46	0.58	0.54	0.53	0.49	0.45
ipTM	0.53	0.52	0.48	0.44	0.43	0.56	0.52	0.50	0.46	0.42
multimer	0.54	0.53	0.48	0.45	0.44	0.56	0.52	0.51	0.47	0.43
Contacts*	187/**20**	180/**0**	190/**0**	139/**86**	126/**83**	185/**3**	101/**99**	143/**151**	134/**126**	154/**5**
PAE < 8 Å#	3431/**72**	1378/**0**	1633/**0**	350/**102**	16/**0**	180/**0**	95/**24**	43/**0**	4/**0**	0/**0**

* Number of contacts (Cβ-Cβ < 8 Å) for the helical regions ($MlaD_{132-263}$) between neighbouring chains. The first number is the average of all i,*i+1* pairs of chains except between the 1st and the last chain (*i* being the index of the chain in the hexamer). The second number (in bold) is between the 1st and the last chain in the hexamer.

# Number of residue pairs with a Predicted Aligned Error (PAE) < 8 Å for the helical regions ($MlaD_{132-263}$) between neighbouring chains. The first number is the average of all *i,i+1* pairs of chains except between the 1st and the last chain (*i* being the index of the chain in the hexamer). The second number (in bold) is between the 1st and the last chain in the hexamer.

Supplementary Table 3: Primers used in this study.

Primer name	DNA sequence
**tamB_3F**	CTTCACTCGCCTGCATTTTACTAATGATACTCCCATACACATTATG
**tamB_3R**	GTTGGTTTAGTGGCGAGCTCCG
**tamB_5F**	GTATAGCCAGCTTGTACCTCAGC
**tamB_5R**	ATTGTGTCCTGTGGATCCACGAGTATGGCTAGAATGTGTAAAC
**mlaD_3F_ery**	ATTATTTAACGGGAGGAAATAATGACTAACACATTTACTCTATATGAGTATGGAGGT
**mlaD_3R**	AGTAACCATCTGGATCAC
**mlaD_5F**	CGTGTATGATTATGGTGC
**mlaD_5R_ery**	GAGAATATTTTATATTTTTGTTCATTTATATATCTCCCATCGCAGATTCGTTAATCG
**ery_F_mod**	ATGAACAAAAATATAAAATATTCTCAAAACTTTTTAACGAGTG
**ery_R_mod**	TTATTTCCTCCCGTTAAATAATAGATAACTATTAAAAATAGACAATACTTG
**catP_F**	GGCCTTTTGCTCACATGTTC
**catP_R**	CCTGAAGTTAACTATTTATCAATTCCTGC
**tetM_F**	AGTAAAATGCAGGCGAGTGAAG
**tetM_R**	GTGGATCCACAGGACACAAT
**mlaE_5F**	GCAGCTACAGCCTTATAC
**mlaE_5R**	TCACTCGCCTGCATTTTACTCAGTGTCTCCCTTTCCC
**mlaD_pRPF185OH_F**	AGCGTTAACAGATCTGAGCTGTACAACAATTTATTCATGGTGAAGCAGAGC
**mlaD_pRPF185OH_R**	TCTCCTTTACTGCAGGAGCTTTAGAATACTTGTTTAATACCGAAATAGGTACCTCCA
**mlaE_pRPF185OH_F**	CGTTAACAGATCTGAGCTACTGTAAGGGAAAGGGAGACACTGTTG
**mlaD_F_pRPF185_2**	CTCTAAGAAAGAAGGAATTCACATGAAGTGGACGACGGAGG
**MCE_R_link_pRPF185_2**	TACCGCTGCCGCTACCACTATTGATAGACTGTAGCATCTTCTCAGTG
**MCE_R_pRPF185_2**	CCAGGAGAGTTGTTGATCACTATTGATAGACTGTAGCATCTTCTCAGTG
**Barrel_F_OmpSP_ pRPF185_2**	CTAAGAAAGAAGGAATTCACATGAAAAAACAATTCGCAACAATGTTAGCAGCAACAGCAGTGTTAGGTGTAACAACAGCATTTGCTCATAATACGGCACAGATTTCAGCTCG

Supplementary Table 4: Strains and plasmids used in this study.

Strain or Plasmid	Description	Antibiotic Resistance	Origin
**SKV38**	Wild-type	/	[1]
**SKV38Δ*mlaD***	Wild-type with chromosomal *mlaD* deletion	Ery	This study
**SKV38Δ*mlaF***	Wild-type with chromosomal *mlaF* deletion	Tc	This study
**SKV38Δ*mlaEFD***	Wild-type with chromosomal *mlaEFD* deletion	Tc	This study
**SKV38Δ*mlaD::mlaD***	Δ*mlaD* complemented with pRPF185::*mlaD*	Ery, Cm	This study
**SKV38Δ*mlaEFD::mlaEFD***	Δ*mlaEFD* complemented with pRPF185::*mlaEFD*	Tc, Cm	This study
**SKV38Δ*mlaD::mlaD*-Barrel-HA**	Δ*mlaD* complemented with pRPF185::*mlaD* *(*Barrel: residues 250-419 with C-ter HA-tag with linker)	Ery, Cm	This study
**SKV38Δ*mlaD::mlaD*-MCE-HA**	Δ*mlaD* complemented with pRPF185::*mlaD* *(TM + MCE domain*: residues 1-155 with C-ter HA-tag with linker)	Ery, Cm	This study
**SKV38Δ*tamB***	Wild-type with chromosomal *tamB* deletion	Tc	This study
**SKV38Δ*mlaD*Δ*tamB***	Wild-type with chromosomal *mlaD* and *tamB* deletion	Ery, Tc	This study
**SKV38Δ*mlaD* (Tn)**	Wild-type with transposon insertion in *mlaD* homologue	Ery	This study
**Δ*mlaD*Δ*tamB* (Tn 27C1)**	Δ*mlaD* with transposon insertion in *tamB*	Ery, Tc	This study
**Δ*mlaD*Δ*tamB* (Tn 90E10)**	Δ*mlaD* with transposon insertion in *tamB*	Ery, Tc	This study
**Δ*mlaD*Δ*sstT* (Tn 5B5)**	Δ*mlaD* with transposon insertion in *sstT*	Ery, Tc	This study
**Δ*mlaD::inter* (Tn 5A4)**	Δ*mlaD* with transposon insertion in intergenic region between *sodA* and FNLLGLLA_01854	Ery, Tc	This study
**Plasmids**			
**pRPF215**	mariner Tn delivery plasmid, P_{tet}::*Himar1* ITR-*ermB*-ITR *catP tetR*	Cm	[2]
**pRPF185**	Tetracycline-inducible expression system fused with β-glucuronidase *gusA* Term(fdx)-P_{tet}-*gusA*-Term(slpA), *catP*	Cm	[3]
**pRPF185Δ*gusA_2***	Tetracycline-inducible expression system with β-glucuronidase *gusA* removed, *catP*	Cm	This study

Supplementary References

1. Knapp, S. *et al.* Natural Competence Is Common among Clinical Isolates of *Veillonella parvula* and Is Useful for Genetic Manipulation of This Key Member of the Oral Microbiome. *Front. Cell. Infect. Microbiol.* **7**, (2017).
2. Dembek, M. *et al.* High-throughput analysis of gene essentiality and sporulation in *Clostridium difficile*. *MBio* **6**, e02383-14 (2015).
3. Fagan, R. P. & Fairweather, N. F. *Clostridium difficile* has two parallel and essential Sec secretion systems. *J. Biol. Chem.* **286**, 27483–27493 (2011).
4. Oliver S. Smart et al. HOLE: A program for the analysis of the pore dimensions of ion channel structural models. *Journal of Molecular Graphics* **14**(6), 354-360 (1996).
5. Letunic, I. & Bork, P. Interactive Tree Of Life (iTOL) v4: recent updates and new developments. *Nucleic Acids Res.* **47**, W256–W259 (2019).
