# Supplementary material for: The Mla system of diderm Firmicute Veillonella parvula reveals an ancestral transenvelope bridge for phospholipid trafficking
Source: Nat Commun. 2023 Nov 23;14:7642. doi: 10.1038/s41467-023-43411-y (PMC10665443; doi:10.1038/s41467-023-43411-y)
Supplement: Supplementary file 3 — Description of Additional Supplementary Files [file 41467_2023_43411_MOESM3_ESM.pdf]

Description of Additional Supplementary Files:

Supplementary Dataset 1: Distribution and accession numbers of Mla components homologues among 1083 genomes representing the bacterial diversity. The presence of MlaB and MlaF is only indicated when they are in cluster with at least one of the other components of the Mla system.

Supplementary Dataset 2: the AlphaFold model of V. parvula MlaD1-130EF with 6:2:2 stoichiometry.

Supplementary Dataset 3: the AlphaFold model of full-length V. parvula MlaD homo-hexamer
