# Supplementary material for: The Mla system of diderm Firmicute Veillonella parvula reveals an ancestral transenvelope bridge for phospholipid trafficking
Source: Nat Commun. 2023 Nov 23;14:7642. doi: 10.1038/s41467-023-43411-y (PMC10665443; doi:10.1038/s41467-023-43411-y)
Supplement: Supplementary file 7 — Reporting Summary [file 41467_2023_43411_MOESM7_ESM.pdf]

nature portfolio

Corresponding author(s): C. Beloin NCOMMS-23-30778-T

Last updated by author(s): Oct 30, 2023

Reporting Summary

Nature Portfolio wishes to improve the reproducibility of the work that we publish. This form provides structure for consistency and transparency in reporting. For further information on Nature Portfolio policies, see our Editorial Policies and the Editorial Policy Checklist.

Statistics

For all statistical analyses, confirm that the following items are present in the figure legend, table legend, main text, or Methods section.

n/a	Confirmed	
☐	☒	The exact sample size (*n*) for each experimental group/condition, given as a discrete number and unit of measurement
☐	☒	A statement on whether measurements were taken from distinct samples or whether the same sample was measured repeatedly
☐	☒	The statistical test(s) used AND whether they are one- or two-sided<br>*Only common tests should be described solely by name; describe more complex techniques in the Methods section.*
☒	☐	A description of all covariates tested
☐	☒	A description of any assumptions or corrections, such as tests of normality and adjustment for multiple comparisons
☐	☒	A full description of the statistical parameters including central tendency (e.g. means) or other basic estimates (e.g. regression coefficient) AND variation (e.g. standard deviation) or associated estimates of uncertainty (e.g. confidence intervals)
☐	☒	For null hypothesis testing, the test statistic (e.g. *F*, *t*, *r*) with confidence intervals, effect sizes, degrees of freedom and *P* value noted<br>*Give P values as exact values whenever suitable.*
☒	☐	For Bayesian analysis, information on the choice of priors and Markov chain Monte Carlo settings
☒	☐	For hierarchical and complex designs, identification of the appropriate level for tests and full reporting of outcomes
☒	☐	Estimates of effect sizes (e.g. Cohen's *d*, Pearson's *r*), indicating how they were calculated

Our web collection on statistics for biologists contains articles on many of the points above.

Software and code

Policy information about availability of computer code

Data collection	AlphaFold v2.3.1 https://github.com/google-deepmind/alphafold<br>Colabfold v1.5.2 https://github.com/sokrypton/ColabFold<br>LocalColabFold v1.5.1 https://github.com/YoshitakaMo/localcolabfold<br>Tomography software (Thermo Fisher Scientific), v.5.2.0.5806REL
Data analysis	Python v2.7 & v3.6 https://www.python.org/<br>DSSP https://swift.cmbi.umcn.nl/gv/dssp/<br>PROSS http://folding.chemistry.msstate.edu/utils/pross.html<br>HMMSEARCH v3.2.1/v3.3.2 package http://hmmer.org/<br>HMMBUILD v3.3.2<br>HOLE v2.2 http://www.holeprogram.org/<br>Chimera v1.15 https://www.cgl.ucsf.edu/chimera/<br>PyMol v2.4.2 https://pymol.org/TopSpin software (Bruker)<br>ImageJv1.53<br>NanoFCM software (NanoFCM Profession V2.0)<br>SMART (Simple Modular Architecture Research Tool)<br>I-TASSER<br>BOCTOPUS2<br>TMHMM 2.0 (Transmembrane Hidden Markov Model)

MacSyFinder2
MAFFT v7.407
BMGE-1.12
IQ-TREE.2.0.6
ModelFinder
iTOLv6.8.1
Prism 9.5.0 (GraphPad Software Inc.)

For manuscripts utilizing custom algorithms or software that are central to the research but not yet described in published literature, software must be made available to editors and reviewers. We strongly encourage code deposition in a community repository (e.g. GitHub). See the Nature Portfolio guidelines for submitting code & software for further information.

Data

Policy information about availability of data

All manuscripts must include a data availability statement. This statement should provide the following information, where applicable:

- Accession codes, unique identifiers, or web links for publicly available datasets
- A description of any restrictions on data availability
- For clinical datasets or third party data, please ensure that the statement adheres to our policy

DATA AVAILABILITY
The authors declare that all data supporting the findings of this study are available within the paper and its supplementary information files. Phylogenomic analysis data are accessible on Mendeley: doi: 10.17632/pj7gmk86cf.1. Source data are provided with this paper.

Research involving human participants, their data, or biological material

Policy information about studies with human participants or human data. See also policy information about sex, gender (identity/presentation), and sexual orientation and race, ethnicity and racism.

Reporting on sex and gender	N/A
Reporting on race, ethnicity, or other socially relevant groupings	N/A
Population characteristics	N/A
Recruitment	N/A
Ethics oversight	N/A

Note that full information on the approval of the study protocol must also be provided in the manuscript.

Field-specific reporting

Please select the one below that is the best fit for your research. If you are not sure, read the appropriate sections before making your selection.

☒ Life sciences ☐ Behavioural & social sciences ☐ Ecological, evolutionary & environmental sciences

For a reference copy of the document with all sections, see nature.com/documents/nr-reporting-summary-flat.pdf

Life sciences study design

All studies must disclose on these points even when the disclosure is negative.

Sample size	no sample-size calculation was performed. For figures were statistics were necessary (Fig. 2, 3 and Supp Fig 2), n was chosen superior or egal to 4. This was sufficient to reach statistical significance with the used Mann Whitney U two-tailed test.
Data exclusions	no data were excluded
Replication	A minimal of three biological replicates were generated providing variance of the results. Results were validated using statistical analyses.
Randomization	NA. No human or animal experiments were performed here, so randomization was not necessary.
Blinding	NA. No human or animal experiments were performed here, so blinding was not necessary.

Reporting for specific materials, systems and methods

We require information from authors about some types of materials, experimental systems and methods used in many studies. Here, indicate whether each material, system or method listed is relevant to your study. If you are not sure if a list item applies to your research, read the appropriate section before selecting a response.

Materials & experimental systems

n/a	Involved in the study
☐	☒ Antibodies
☒	☐ Eukaryotic cell lines
☒	☐ Palaeontology and archaeology
☒	☐ Animals and other organisms
☒	☐ Clinical data
☒	☐ Dual use research of concern
☒	☐ Plants

Methods

n/a	Involved in the study
☒	☐ ChIP-seq
☐	☒ Flow cytometry
☒	☐ MRI-based neuroimaging

Antibodies

Antibodies used

Commercial antibodies
- secondary goat HRP-linked anti-rabbit antibody (Abcam #98431) at 1:10,000 dilution
- primary anti-HA tag polyclonal HRP-linked antibody (Novus Biologicals, ref. NB600-363) at 1:4000 dilution

'In house' antibodies
- our own generated polyclonal rabbit anti-MlaD serum (CovaLab) used at 1:4000 dilution (raised against V. parvula full-length MlaD recombinantly expressed in E. coli BL21(DE3))
- rabbit polyclonal antisera against E. coli TolC and SecA gifted from Dr Philippe Delepelaire at 1:8000 dilution

Validation

The antibodies used in the study were validated by performing the experiments for which they were purchased (western blot) and observing the presence of the markage when the HA-tagged target was expressed and its absence in the same strain but containing an empty expression vector and/or a vector expressing the untagged version of the protein. Please note that the commercial antibodies have also been independently validated by its suppliers. Anti-MlaD, -SecA and -TolC were validated using corresponding mutants.

Flow Cytometry

Plots

Confirm that:

☐ The axis labels state the marker and fluorochrome used (e.g. CD4-FITC).

☐ The axis scales are clearly visible. Include numbers along axes only for bottom left plot of group (a 'group' is an analysis of identical markers).

☐ All plots are contour plots with outliers or pseudocolor plots.

☐ A numerical value for number of cells or percentage (with statistics) is provided.

Methodology

Sample preparation

Describe the sample preparation, detailing the biological source of the cells and any tissue processing steps used.

Instrument

Identify the instrument used for data collection, specifying make and model number.

Software

Describe the software used to collect and analyze the flow cytometry data. For custom code that has been deposited into a community repository, provide accession details.

Cell population abundance

Describe the abundance of the relevant cell populations within post-sort fractions, providing details on the purity of the samples and how it was determined.

Gating strategy

Describe the gating strategy used for all relevant experiments, specifying the preliminary FSC/SSC gates of the starting cell population, indicating where boundaries between "positive" and "negative" staining cell populations are defined.

☐ Tick this box to confirm that a figure exemplifying the gating strategy is provided in the Supplementary Information.
