# Supplementary material for: The Mla system of diderm Firmicute Veillonella parvula reveals an ancestral transenvelope bridge for phospholipid trafficking
Source: Nat Commun. 2023 Nov 23;14:7642. doi: 10.1038/s41467-023-43411-y (PMC10665443; doi:10.1038/s41467-023-43411-y)
Supplement: Supplementary file 8 — Source Data [file 41467_2023_43411_MOESM8_ESM.zip › Source data/Source_data2_Main_and_SuppFigures.pdf]

Raw data for Mla paper:
Data supporting **main text figures**

Fig 2

Fig 2: Phenotypic characterisation of Δ*mla* strains

A

Regular media (SK)

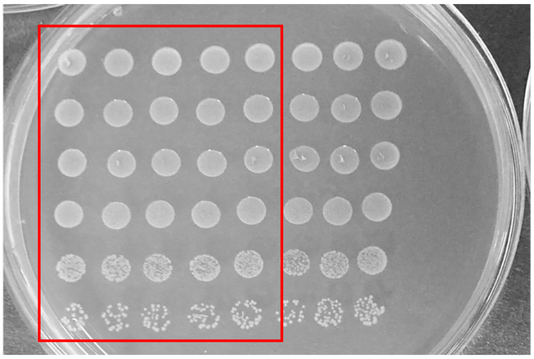

WT ΔEFD ::EFD ΔD::D (ΔmlaD::mlaD, 250ng aTc) (ΔmlaEFD ::mlaEFD, 250ng aTc) (ΔmlaEFD ::mlaEFD, 300ng aTc)

SDS 0.004% + EDTA 312.5uM

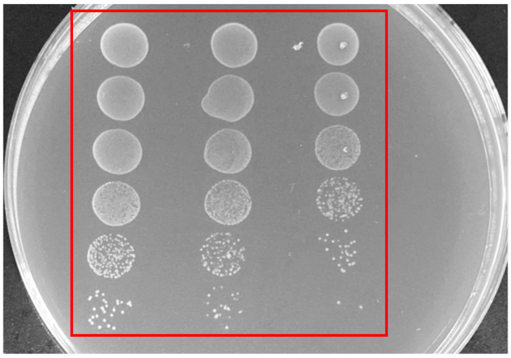

WT ΔEFD ::EFD ΔD ::D

Vancomycin 250ug/ul

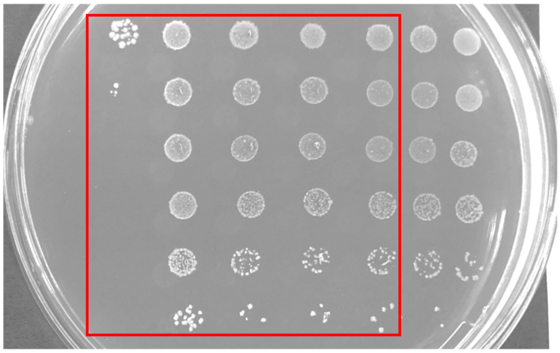

WT ΔEFD ::EFD ΔD ::D (ΔmlaD::mlaD, 250ng aTc) (ΔmlaEFD ::mlaEFD, 250ng aTc)

Fig 2A: Efficiency of plating assays to assess OM permeability of WT and Δ*mla* mutants and complemented Δ*mlaD::mlaD* / Δ*mlaEFD::mlaEFD* strains

Attempted higher concentrations of aTc induction to see if rescue of vancomycin resistance was possible

B

SDS 0.004% + EDTA 312.5uM

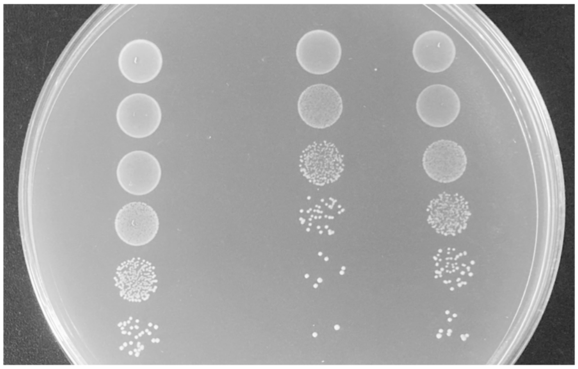

WT ΔD ΔD:185 ΔD:D ΔEFD :EFD

SDS 0.004% + EDTA 312.5uM

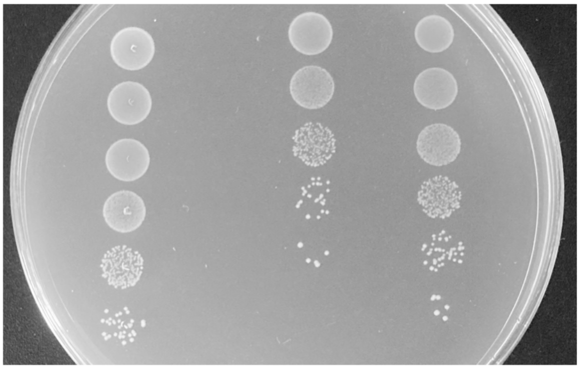

WT ΔD ΔD:185 ΔD:D ΔEFD :EFD

Fig 2B: aTc-induced Δ*mlaD* and Δ*mlaEFD* containing empty vector (pRPF185) also plated to test for any effect; harbouring empty plasmid does not affect growth / complementation

- Red font represents strains induced with aTc
- Data not included in manuscript

Fig 2: OMV production of WT / Δ*mla* / Δ*mla::mla* strains

C i)

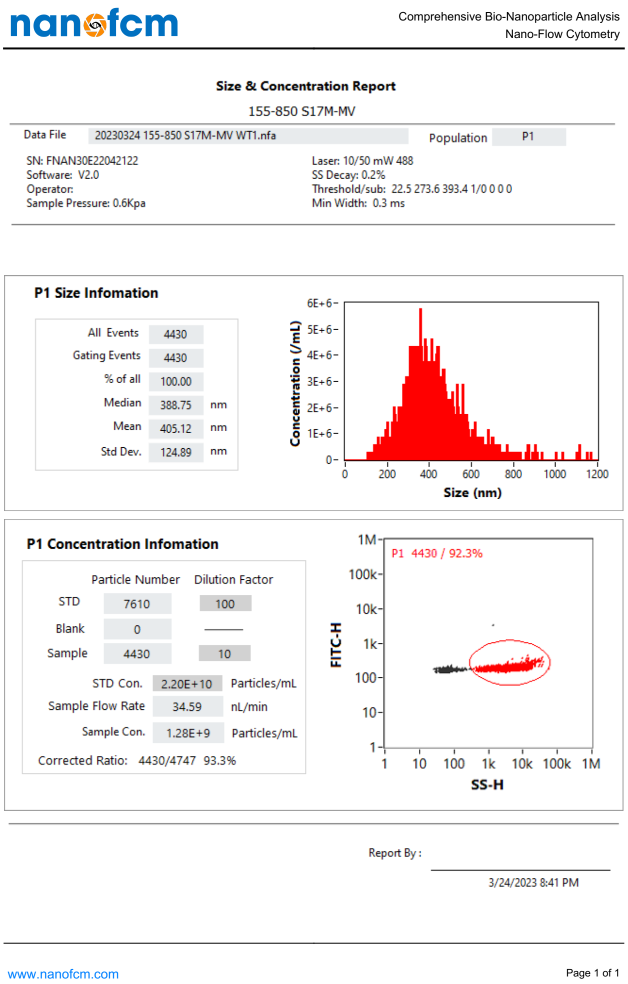

C ii)

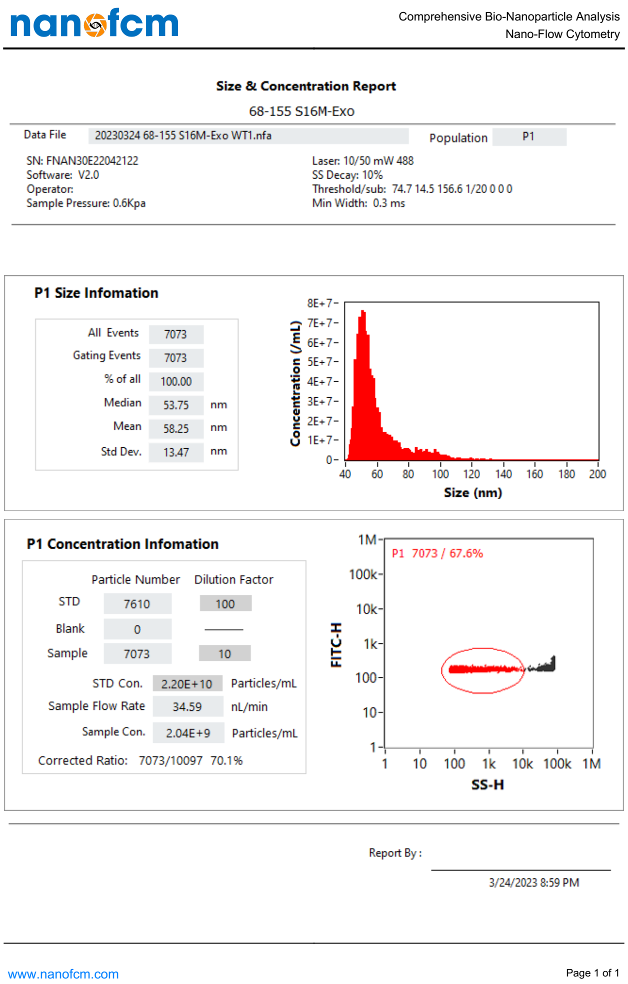

Fig 2C: NanoFCM-generated reports showing total count and gating strategy

C i) Gating for 'large events' (*V. parvula* cells) showing average size of ~400nm
C ii) Gating for 'small events) (outer membrane vesicles) showing average size of ~60nm

In the case of WT quantification, the same 1/10 dilution could be used to quantify both large and small events. For hypervesiculating strains, generally a 1/10 dilution was used for large event (cell) counts, whilst a 1/100 dilution was used for small event (OMV) counts.

For full counts, see spreadsheet with all cell / OMV data for each strain.

Fig 2: TLC of OMV lipid extracts (WT vs Δ*mlaD)*

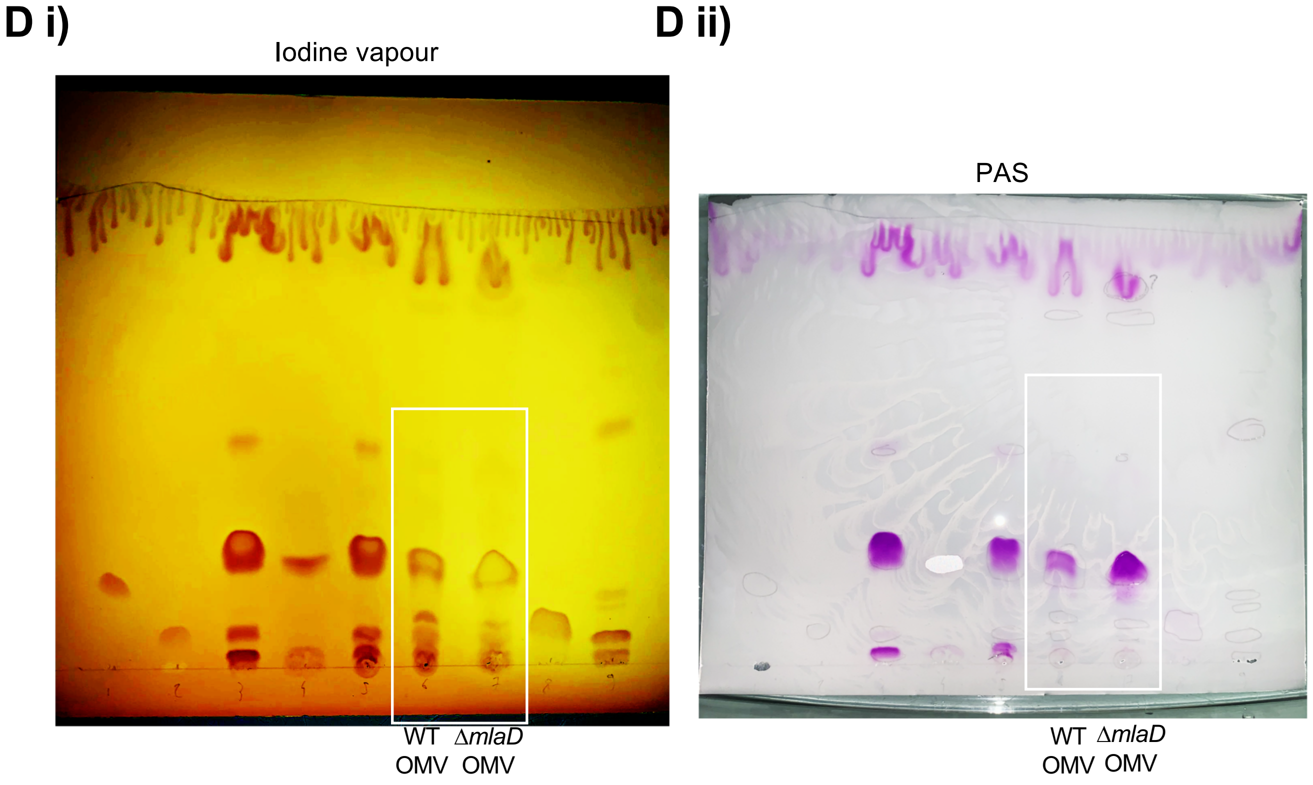

Fig 2D: Full TLC plates showing lipid extract migrations of WT and Δ*mlaD* OMVs (highlighted in white boxes) stained by iodine vapour **(Di)** and Periodic Acid Schiff (PAS) reagent **(Dii).**
Other samples on plate are being used for a further publication

Fig 3

Fig 3: TamB is a suppressor of the Δ*mlaD* phenotype

A

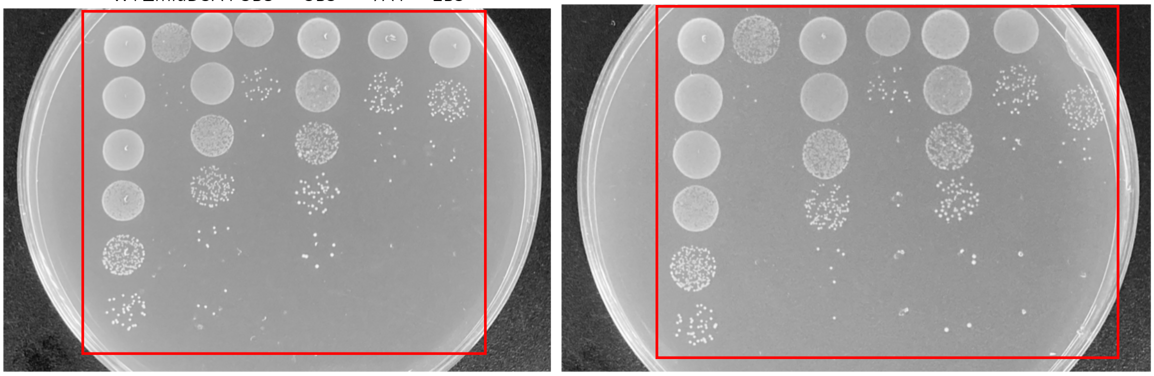

Fig 3A: Serial dilution plating of all 10 Tn-insertion suppressor mutants of Δ*mlaD.* As shown in the paper, only 4 Tn insertions substantially rescued the detergent sensitivity phenotype of Δ*mlaD.* The red boxes highlight the area presented in Main Fig 3B.

B

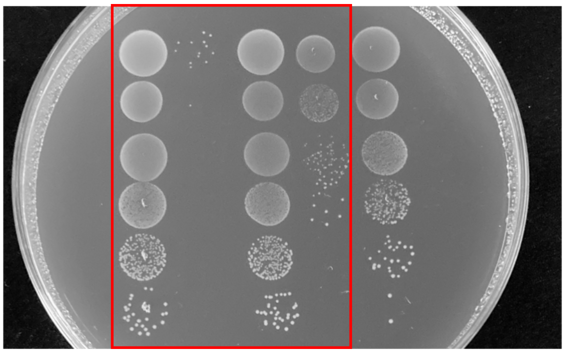

Fig 3B: Serial dilution plating of clean deletion mutants of Δ*tamB,* Δ*mlaD* and Δ*mlaD*Δ*tamB.* The red box highlights the portion of the plate that is used in Main Fig 3C. Additional efficiency of plating assays of these mutants are included in Supplementary Figure 4 to highlight the slight increase in vancomycin sensitivity upon deletion of *tamB,* and the extend of phenotypic rescue in Δ*mlaD*Δ*tamB.*

Fig 4

Fig 4D: MlaD localisation (Δ*mlaD*::MCE domain)

A

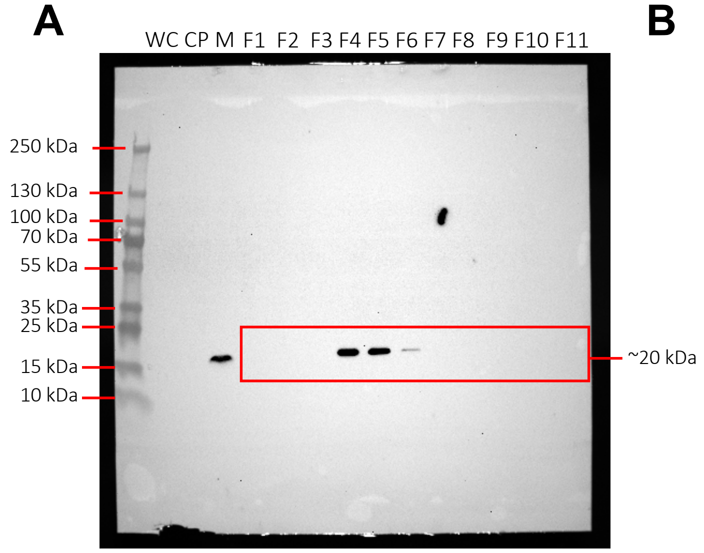

B

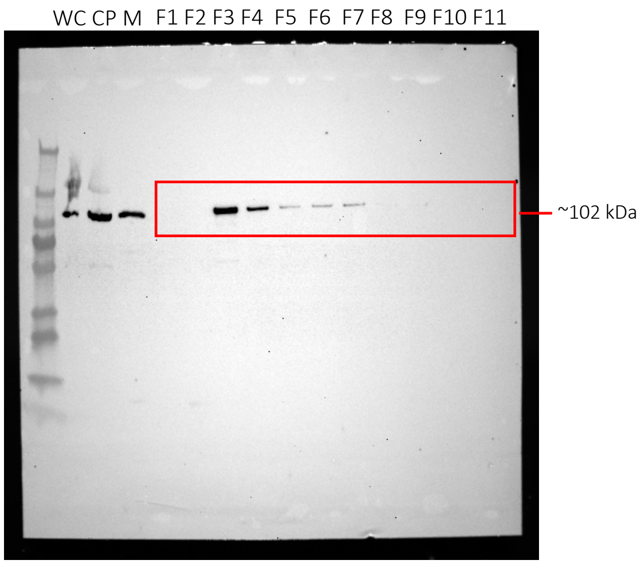

C

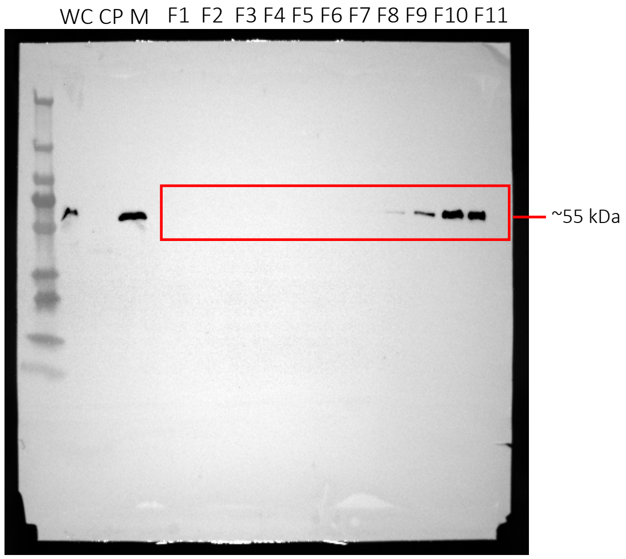

Fig 4D: Immunoblot of Δ*mlaD*::TMD-MCE membrane fractions. Red box highlights data used for Fig 4D.

A) Anti-HA stained membrane fractions
B) Anti-SecA stained membrane fractions
C) Anti-TolC stained membrane fractions

WC = whole cell lysate
CP = cytoplasmic / periplasmic fraction
M = membrane pellet (OM / IM)

Fig 4D: MlaD localisation (Δ*mlaD*::Barrel domain)

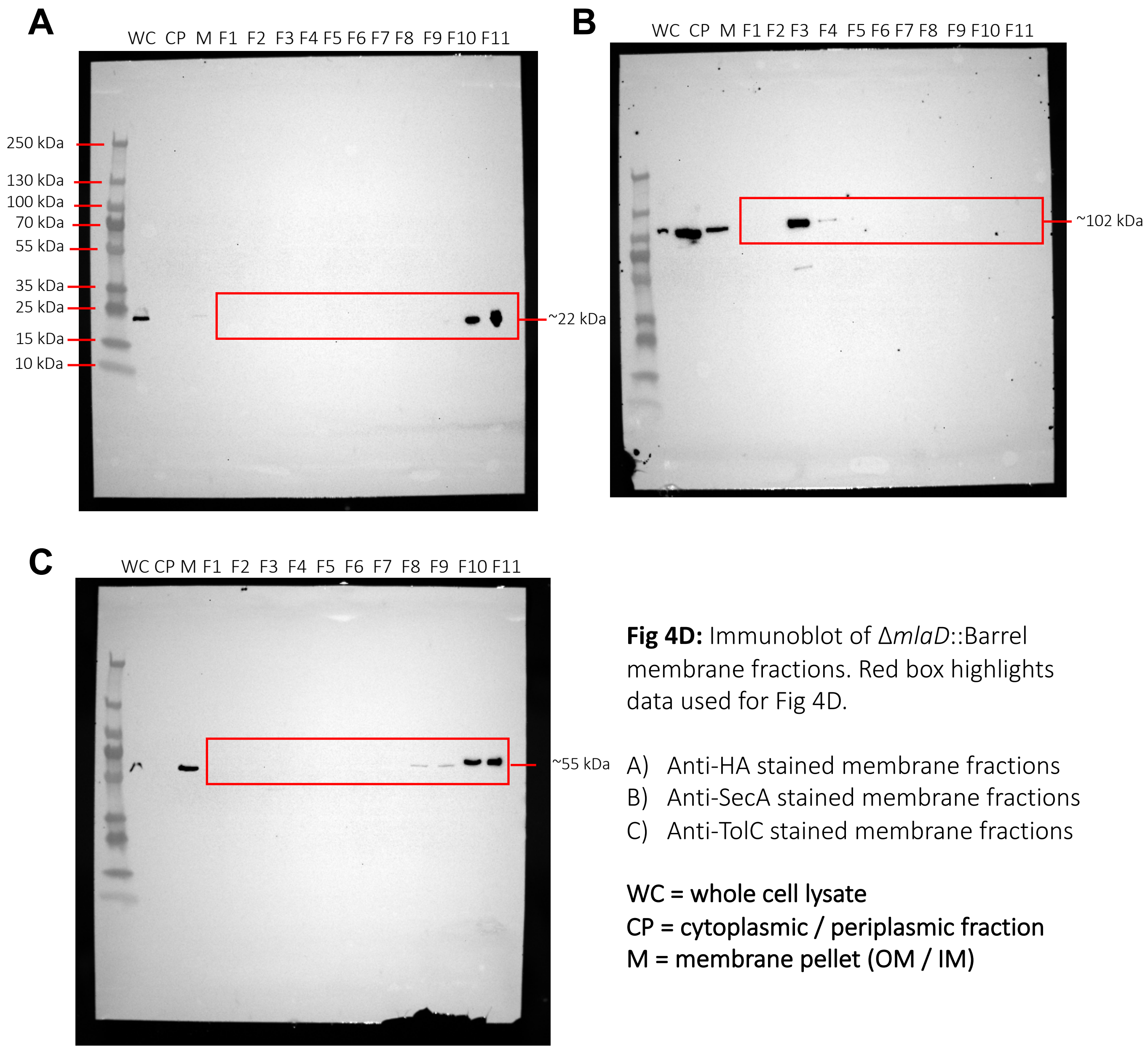

Fig 4D: Immunoblot of Δ*mlaD*::Barrel membrane fractions. Red box highlights data used for Fig 4D.

A) Anti-HA stained membrane fractions
B) Anti-SecA stained membrane fractions
C) Anti-TolC stained membrane fractions

WC = whole cell lysate
CP = cytoplasmic / periplasmic fraction
M = membrane pellet (OM / IM)

Fig 4D: MlaD localisation (WT; full-length MlaD)

A

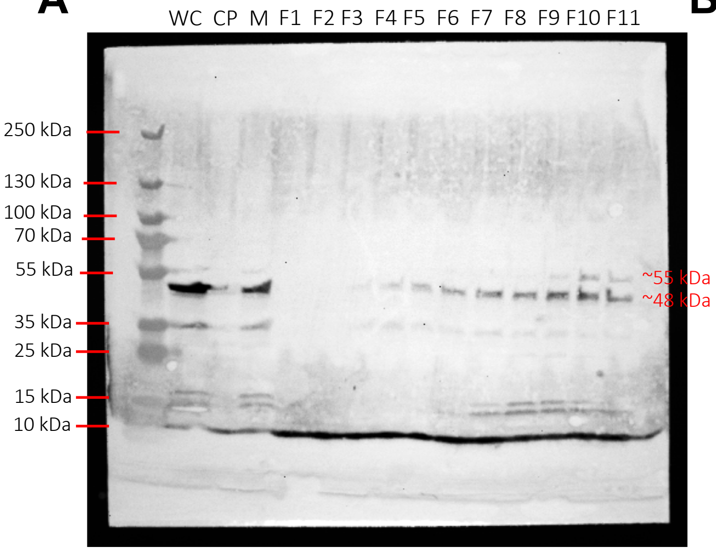

B

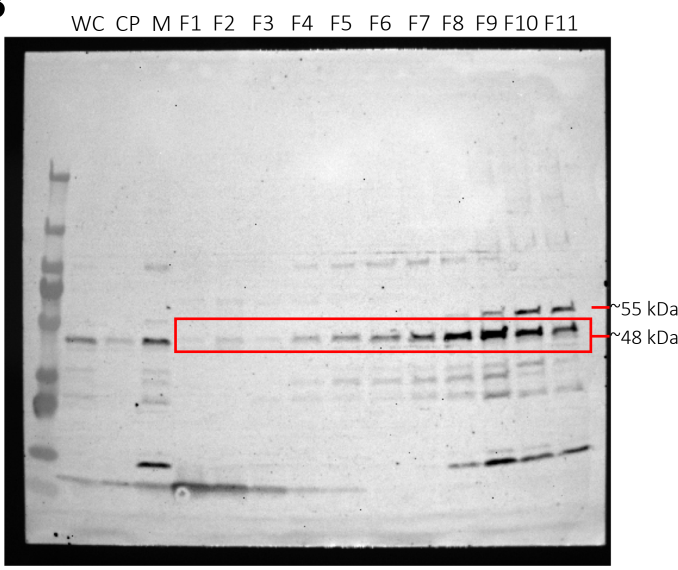

C

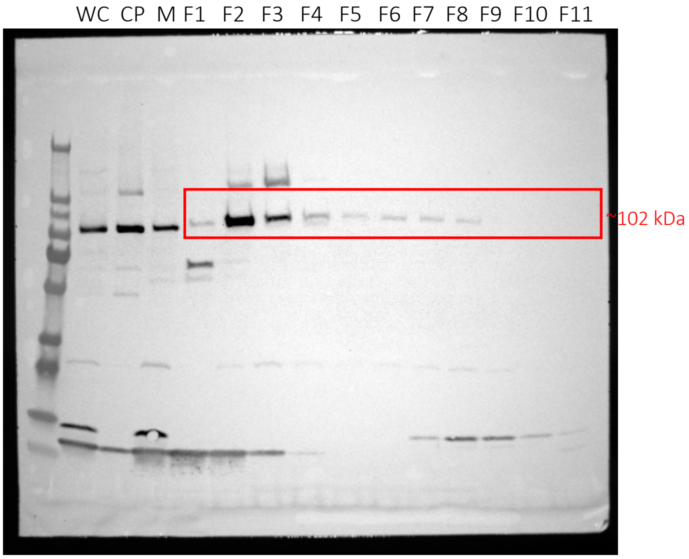

Fig 4D: Immunoblot of WT SKV38 fractions. Red box highlights data used for Fig 4D.

A) Bio rep1: Anti-mlaD stained membrane fractions (shows MlaD (48 kDa) and TolC (55 kDa))
B) Bio rep2: Anti-mlaD stained membrane fractions (shows MlaD (48 kDa) and TolC (55 kDa))
C) Anti-SecA stained membrane fraction of Bio rep2

WC = whole cell lysate
CP = cytoplasmic / periplasmic fraction
M = membrane pellet (OM / IM)

Raw data for Mla paper:
Data supporting **supplementary figures**

Supplementary Fig 2: Phenotypes of Δ*mla* strains

A

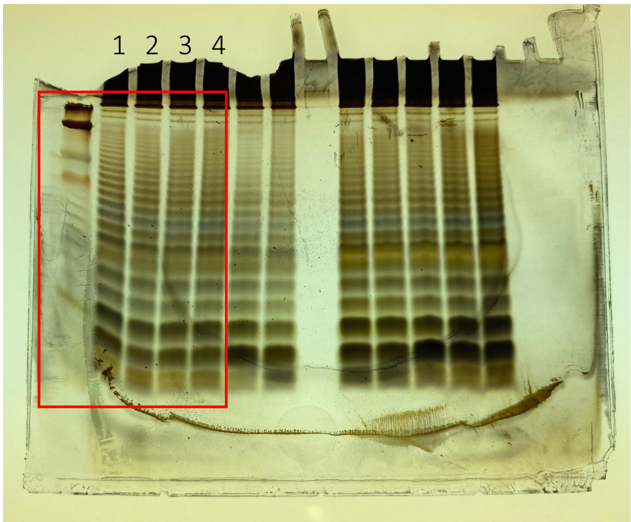

(corresponding to Supp Fig 2B)

LPS extracts from WT and Δ*mla* strains were run on SDS-PAGE and silver-stained (see methods). No changes in LPS laddering (overall profile) or quantity were observed.

1) WT
2) Δ*mlaD*
3) Δ*mlaF*
4) Δ*mlaEFD*

B

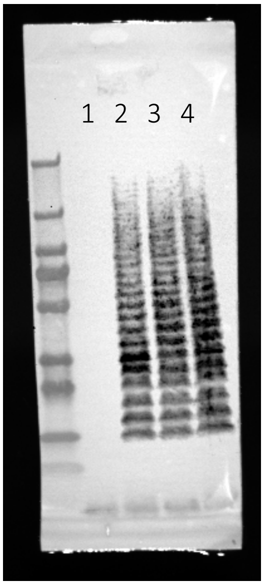

Additional: LPS extracts from WT and Δ*mlaD* strains were run on SDS-PAGE and stained via immunoblot with a whole-cell antibody developed against WT *V. parvula* SKV38. No changes in LPS laddering (overall profile) or quantity were observed. LPS mutants (lanes 1 + 2) were used as controls.

1) LPS mutant 1 (no O-antigen)
2) LPS mutant 2 (putative lipid A-core modification)
3) Δ*mlaD*
4) WT

Supporting data; data not included in Supp figure 2.

Supplementary Fig 3: PL composition of WT SKV38

A

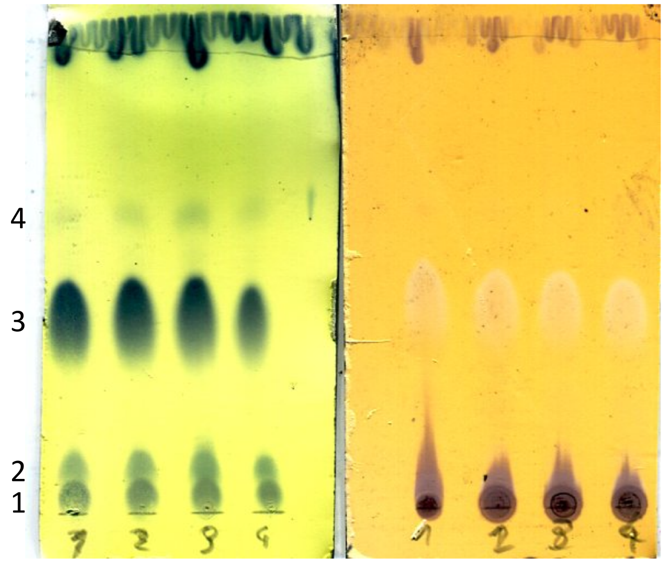

B

Relative proportion (%) of each lipid species			
**1 (PG)**	**2**	**3 (PE)**	**4**
17.51	9.9	68.56	4.02
25.47	8.09	63.57	2.85
19.28	7.73	69.75	3.23
19.11	8.74	68.22	3.91

Supp Fig 3A: Raw data corresponding to Supplementary Figure 3B: **Relative proportion of each lipid species in WT SKV38.**
A) 4 biological replicates of whole-cell WT lipid extracts were run via TLC and stained with phosphomolybdic acid. The 4 major lipids present in the envelope of *V. parvula* SVK38 are numbered on the y-axis, 1-4. Lipid 1 has been identified as PG, whilst lipid 3 is PE. **B)** The relative proportion of each lipid species was quantified by ImageJ; n = 4.

Supplementary Fig 6: Permeability phenotypes of *ΔmlaD / ΔtamB / ΔmlaDΔtamB*

A

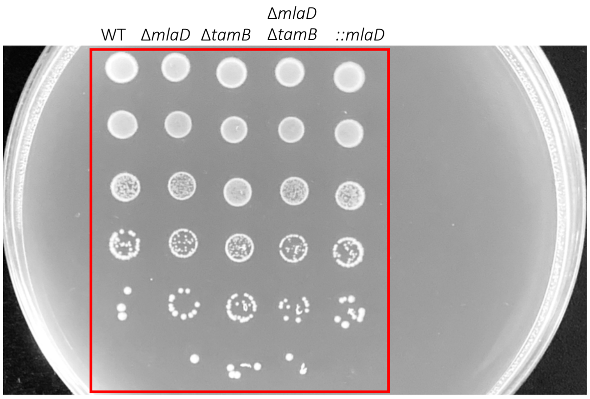

SK (control)

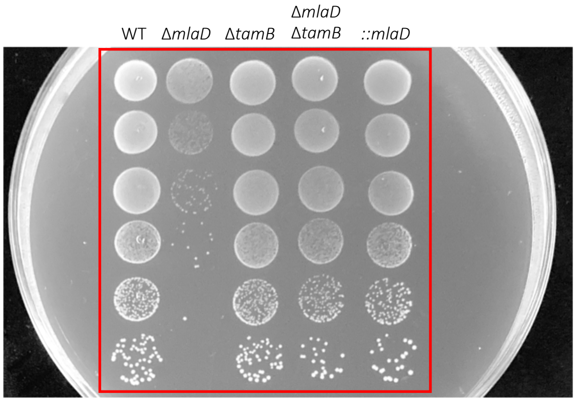

SDS 0.003% / EDTA 312.5µM

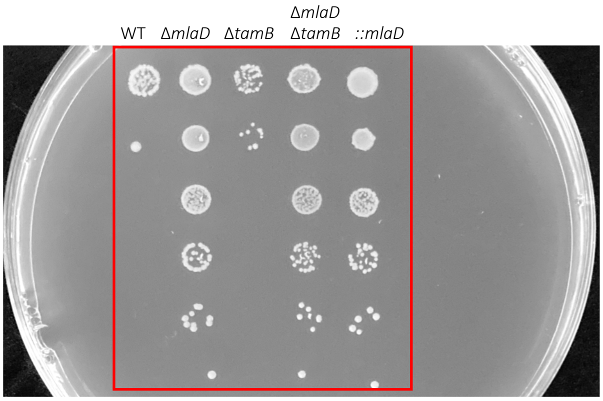

Vancomycin 250µg/ml

Fig S6A: Serial dilution plating of WT, *ΔmlaD, ΔtamB, ΔmlaDΔtamB* and *ΔmlaD::mlaD* strains. The red boxes highlight the area presented in Fig S4A.

B

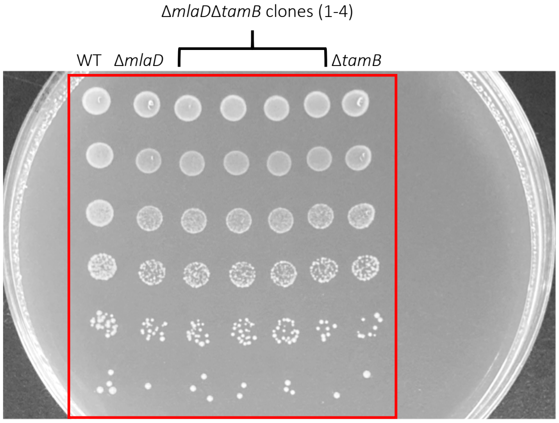

SK (control)

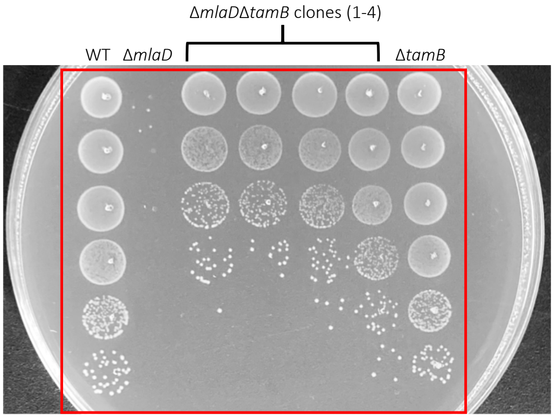

SDS 0.004% / EDTA 312.5µM

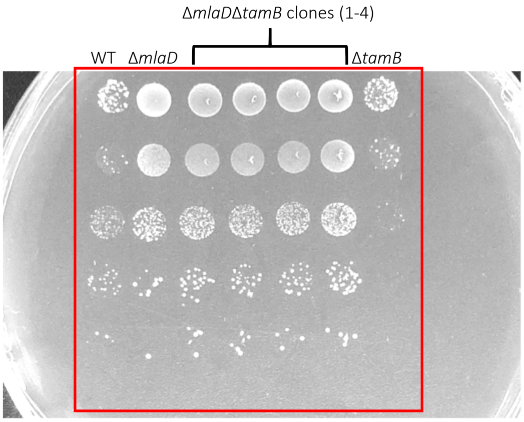

Vancomycin 250µg/ml

Fig S6B: Serial dilution plating of WT, *ΔmlaD, ΔtamB* and 4 biological replicates of *ΔmlaDΔtamB.* The red boxes highlight the area presented in Fig S4B.
